# Psychometric evaluation of the Comprehensive Autistic Trait Inventory in autistic and non-autistic adults

**DOI:** 10.1177/13623613251347740

**Published:** 2025-07-16

**Authors:** Michael CW English, Rebecca E Poulsen, Murray T Maybery, David McAlpine, Paul F Sowman, Elizabeth Pellicano

**Affiliations:** 1 The University of Western Australia, Australia; 2 The Kids Research Institute Australia, Australia; 3 Macquarie University, Australia; 4 Auckland University of Technology, New Zealand; 5 University College London, UK

**Keywords:** autistic traits, factor analysis, measurement, psychometric analysis, screening

## Abstract

Measures of autistic traits are only useful – for pre-diagnostic screening, exploring individual differences, and gaining personal insight – if they efficiently and accurately assess autism as currently conceptualised while maintaining psychometric validity across different demographic groups. We recruited 1322 autistic and 1279 non-autistic adults who varied in autism status (non-autistic, diagnosed autistic, self-identifying autistic) and gender (cisgender men, cisgender women, gender diverse) to assess the psychometric properties of the Comprehensive Autistic Trait Inventory, a recently developed measure of autistic traits that examines six trait domains using 42 self-report statements. Factor fit for the six subscales was appropriate, as was total-scale and subscale reliability. Importantly, measurement invariance was demonstrated based on both autism status and gender, indicating that Comprehensive Autistic Trait Inventory scores of these group members can be directly compared. Autistic traits were highly similar between diagnosed and self-identifying autistic adults, while gender-diverse participants showed more autistic traits than their cisgender counterparts. A total-scale discrimination threshold of 147.5 calculated was suggested which corresponded to sensitivity and specificity of 77.20 and 87.41, respectively. Our analysis indicates that the Comprehensive Autistic Trait Inventory is a practical measure of autistic traits in non-autistic and autistic participants that is useful for researchers and clinicians and for affirming self-identity.

**Lay abstract:**

The Comprehensive Autistic Trait Inventory (CATI) is a free questionnaire designed to measure autistic traits in both autistic and non-autistic adults. The CATI includes 42 items focusing on six areas: Social Interactions, Communication, Social Camouflage, Self-Regulating Behaviours, Cognitive Flexibility, and Sensory Sensitivity. Here, we set out to determine whether the CATI can accurately measure autistic traits in both autistic (both diagnosed and self-identifying) and non-autistic people, as well as people of different genders. We also wanted to explore the extent to which trait scores differed between these groups of individuals. Our study recruited over 2600 participants, including 1322 autistic and 1279 non-autistic adults. Our findings suggest that the CATI works the way it was designed to. It is a reliable and accurate tool for measuring autistic traits, can distinguish between autistic and non-autistic people, and appears appropriate for people of different genders. Notably, we found that people who self-identify as autistic have similar trait scores to those with a clinical diagnosis of autism and that gender-diverse people scored higher on autistic traits compared to cisgender people. Our data suggest that the CATI is a useful tool for measuring autistic traits in autistic and non-autistic people and for understanding the way that autistic people vary from one another. It should be helpful for researchers and clinicians, and support a public understanding of autism.

Autism is a neurodevelopmental divergence affecting people across the lifespan (Kapp et al., 2013; Silberman, 2015). In current diagnostic handbooks, autism is characterised by atypical social interaction and communication, as well as repetitive behaviours, sensory differences and an insistence on sameness (American Psychiatric Association [APA], 2022). Qualitatively similar characteristics are reported in the non-clinical population, with ‘sub-threshold’ autistic traits observed in parents of autistic children (Bailey et al., 1998; Kanner, 1944; Rutter, 2000; Sucksmith et al., 2011) and, more broadly, normally distributed within the general population (Baron-Cohen et al., 2011; Ruzich et al., 2015). Supporting the notion of an autism continuum that extends into non-clinical populations, large-scale studies suggest an overlapping genetic aetiology for autism and autistic traits that is evidenced through twin concordance (Lundström et al., 2012) and common (Bralten et al., 2018; Robinson et al., 2016) and *de novo* (Robinson et al., 2016) genetic variance.

Self-report autistic-trait questionnaires have facilitated research on the autistic continuum and bring several advantages to assessing autistic traits in clinical and non-clinical adult populations (for review, see Lyall, 2023). Theoretical models linking autistic characteristics to particular psychological sequelae can be evaluated by sampling the general population (Johnston et al., 2013; Mosner et al., 2019), easing the recruitment of large samples and avoiding the need to control for the substantial number of co-occurring conditions typically reported for formally diagnosed autistic groups (Jarrold & Brock, 2004; Landry & Chouinard, 2016). Trait-based studies provide accessible preliminary evidence that can inform studies exploring clinical populations. Furthermore, though trait dimensions often align with diagnostic criteria (e.g. communication difficulties), there is growing recognition of the diverse presentation of autism (Belcher et al., 2022; Lundström et al., 2012; Sasson & Bottema-Beutel, 2022). Self-report surveys can reveal emerging traits not currently used in formal diagnosis (e.g. social camouflaging; (Adkin, 2023; Chahboun et al., 2022; Lundin Remnélius & Bölte, 2023; Miller et al., 2021; Pearson & Rose, 2021; Sucksmith et al., 2011).

Finally, an increased need exists for assessing autistic traits in the general population using psychometrically accurate screeners, especially as demand for diagnostic services increases for both children and adults (L. Jones et al., 2014; Levy et al., 2009; McKenzie et al., 2015). This latter advantage is important because access to autism assessments is not equitable. Certain groups are particularly affected by a dearth of services, including cisgender women, gender minority groups, people with intellectual disabilities, individuals with limited financial means (or support), and those who live in underserviced regions (Anderson et al., 2018; Dababnah et al., 2018; Davis et al., 2022; Lockwood Estrin et al., 2021; Malik-Soni et al., 2022). When access to formal diagnosis is unavailable (for either child or adult) or otherwise undesired (Lewis, 2017), self-reported measures of autistic traits can help people embrace an autistic identity and, if desired, navigate towards a formal diagnosis (Botha et al., 2020; Crawshaw, 2023; Davies et al., 2023).

## Concerns with established measures of autistic traits

Given their potential benefits, several self-report surveys and questionnaires have been developed to quantify autistic traits in adults. These vary in length (i.e. number of items) and the breadth of dimensions captured. As the understanding and exploration of autism has evolved (Conner et al., 2019; Gunderson et al., 2023; Murray et al., 2005), with diagnostic criteria updated accordingly, once commonly employed measures have become outdated (Agelink van Rentergem et al., 2019; Ashwood et al., 2016; Conner et al., 2019; Dorlack et al., 2018; Yu et al., 2023). For example, broad measures of autistic traits, such as the Broad Autism Phenotype Questionnaire (BAPQ; Hurley et al., 2007), Autism-Spectrum Quotient (AQ; Baron-Cohen et al., 2001) and Social Responsiveness Scale (SRS-A; Constantino, 2012), have too few, if any, items to appropriately assess differences in sensory sensitivity. The Ritvo Autism Asperger Diagnostic Scale–Revised (RAADS-R; Ritvo et al., 2011) partially addresses this issue, but the combined ‘Sensory Motor’ subscale limits the specificity of the results. Other scales (e.g. AQ, SRS-A, RAADS-R) focus heavily on social rather than non-social characteristics, limiting the representation of non-social autistic features in research and screening processes. For example, while the RAADS-R examines autistic traits across four subscales, the ‘Social Relatedness Problems’ subscale makes up 39 of the total 80 items, disproportionately contributing towards the total-scale score (Ritvo et al., 2011).

Of further concern is the statistical properties of many autistic trait measures. An influential paper by Nishiyama et al. (2014) compared the psychometric properties of most trait measures available at the time, with some of the most prominent measures (e.g. AQ, SRS-A) showing troubling internal consistency reliability and discriminant validity. Others are comprised of subscales with poor reliability (e.g. *Imagination* in the AQ; Austin, 2005) and/or have factor models not replicated in independent analyses (Constantino et al., 2003; English et al., 2020; Sturm et al., 2024). Most scales have shown questionable screening properties when applied to clinical settings (for review, see Wigham et al., 2019). For example, the RAADS-R showed no predictive ability in identifying whether 50 adult users of a UK National Health Service specialist autism service would go on to receive an autism diagnosis (S. L. Jones et al., 2021). Similar outcomes are reported for the AQ and SRS-A (Adamou et al., 2021; Ashwood et al., 2016; Bezemer et al., 2021).

Finally, terms and language used in some measures can stigmatise and reinforce outdated stereotypes (e.g. ‘I am fascinated by dates’, ‘I would rather go to the theatre than a museum’; Agelink van Rentergem et al., 2019). The language used to describe autistic traits is increasingly important when considering the development of a questionnaire since ableist, non-inclusive or stigmatising phrasing can lead to relational ‘ruptures’ between test-takers – who are often autistic people – and test administrators, researchers and clinicians – who may not be autistic (Bottema-Beutel et al., 2021; S. C. Jones, 2022). This issue is one best addressed by involving autistic people in the design, acquisition and analysis of research (Fletcher-Watson et al., 2019; Nicolaidis et al., 2011; Poulsen et al., 2022). Co-design methodology is demonstrated to ensure research outcomes better align with research priorities identified by the autistic community (Pellicano et al., 2021).

## The Comprehensive Autistic Trait Inventory

The Comprehensive Autistic Trait Inventory (CATI) is a recently developed self-report measure of autistic traits that aims to overcome many of the concerns related to outmoded or outdated assessments (English et al., 2021). It achieves this by (1) including domains measuring social camouflaging and sensory sensitivities and (2) involving key stakeholders – autism researchers, clinicians and autistic adults – in its development. Development of the CATI required a thorough psychometric process spread across three studies. In the first study (Study 1), 1119 participants recruited from the general population were presented with 107 pilot items from which exploratory factor analysis reduced the final set to 42, spread evenly across six subscales labelled *Social Interactions, Communication, Social Camouflage, Repetitive Behaviours, Cognitive Rigidity* and *Sensory Sensitivity*. In Study 2, the factor structure was validated in an additional set of 1068 individuals and found to be measurement invariant between cisgender men and women. Study 2 also demonstrated that the CATI outperformed the existing AQ and BAPQ measures both in terms of internal consistency, reliability and the classification of autistic and non-autistic participants (English et al., 2021). Finally, in Study 3, convergent validity was confirmed for each of the six CATI subscales, with the scores for each subscale correlating strongly with a conceptually similar established measure. While these outcomes are encouraging and demonstrate that the CATI presents a reliable and comprehensive tool for assessing autistic traits in the general population, the lack of analyses using a sizable autistic sample means it remains to be determined whether it is well suited for reliably measuring traits in *autistic people*.

## The current study

Here, we aimed to investigate the psychometric properties of the CATI and its associated clinical utility in autistic adults beyond the preliminary assessment conducted by English et al. (2021). In addition, we set out to determine whether the CATI is consistently reliable across different genders. Finally, we set out to determine the CATI’s reliability among both clinically diagnosed and self-identifying autistic adults. To address these aims, we combined data across four separate datasets to generate large samples of autistic and non-autistic people. To test the CATI’s psychometric properties, we conducted a series of analyses comparing autistic and non-autistic people, as well as diagnosed autistic and self-identifying autistic people. These analyses included confirmatory factor analyses (CFAs), measurement invariance testing and internal consistency reliability examinations. To test the CATI’s clinical utility, we examined group differences across the total-scale and subscale scores, assessed sensitivity and specificity for classifying autistic and non-autistic people, and identified an optimal total-score threshold for classification. Finally, we examined how gender interacts with the trait profiles of autistic and non-autistic participants to assess the appropriateness of the CATI for different genders.

## Method

### Participants

We combined datasets from surveys that administered the CATI to both non-autistic and autistic people, including English et al. (2021; Study 2, Sample 1; *n* = 1113), Brett et al. (2024; Study 2, Sample 2; *n* = 268) and Poulsen et al. (2025, Sample 3; *n* = 381). We further supplemented these datasets with additional data collection (Sample 4; *n* = 839) to increase the representation of autistic (formally diagnosed and self-identifying) people, balance gender representation within the autistic groups and improve the general representation of gender-diverse people. Ethical approval was received to recruit participants in the Sample 4 dataset from the University of Western Australia’s Human Research Ethics Office (2019/RA/4/20/5546), and written consent was obtained from each participant. Similar ethical approvals for data collection and analysis were also obtained for the pre-existing datasets prior to data collection.

The pooled dataset consisted of 2601 participants (for descriptive statistics, see Table 1; for individual datasets, see Table S1 in Supplemental Materials), including participants who reported (1) being autistic with a formal diagnosis (*n* = 737; ‘Autistic _DX_’), (2) self-identified as autistic, but without an accompanying formal diagnosis (*n* = 585; ‘Autistic _SELF_’), and (3) being non-autistic (*n* = 1279). For statistical comparisons, we occasionally combined the diagnosed and self-identifying autistic groups in our analyses (*n* = 1322; ‘Autistic _ALL_’). All participants were recruited either via Prolific Academic (Samples 1, 2, and 4) or advertising on social media (Sample 3) and completed the CATI and demographics-related questions online using survey software (Qualtrics or REDCap). Where a participant recruited via the Prolific Academic platform contributed to multiple datasets (detected via unique user identifiers), the more recent participant record was retained. Participants completed the CATI using consistent item ordering and the response options outlined in English et al. (2021).^1^

**Table 1.** Demographic information for participants, including age, autistic identity, and gender identity.

	All participants	Non-autistic	Autistic _ALL_	Autistic _DX_	Autistic _SELF_
**Total N**	2601 (100%)	1279 (49.2%)	1322 (50.8%)	737 (28.3%)	585 (22.5%)
**Gender**					
Cisgender man	1205 (46.3%)	645 (24.8%)	560 (24.8%)	312 (12.0%)	248 (9.5%)
Cisgender woman	1059 (40.7%)	556 (21.3%)	503 (19.3%)	297 (11.4%)	206 (7.9%)
Gender diverse^a^	336 (12.9%)	78 (3.0%)	258 (9.9%)	127 (4.9%)	131 (5.0%)
Gender not given	1 (0.0%)	0 (0.0%)	1 (0.0%)	1 (0.0%)	0 (0.0%)
**Mean Age [*SD*]**	36.1 [11.8]	37.6 [12.4]	34.8 [11.1]	34.1 [10.7]	35.7 [11.5]

Numbers in parentheses refer to the percentage of the total sample, except for age, where the numbers refer to the standard deviation.

a Gender diverse is inclusive of identities such as, but not limited to, nonbinary, genderqueer, demigender, agender and trans (i.e. participants whose gender identity does not ‘align’ with their sex assigned at birth). See Table S3 in Supplemental Materials for further details.

Participant recruitment varied between specific samples. All participants were required to speak English. For samples collected via Prolific Academic (i.e. Samples 1, 2 and 4), participants were restricted mainly to people from the United Kingdom, the United States, Australia, Canada and New Zealand, and were remunerated for their participation. Sample 3 had no location restriction and no remuneration. All participant responses were screened for atypically fast completion times and response patterns, which indicate fabrication. Participants in Samples 1 and 4 were also subject to attention checks in the form of items integrated into parts of the online survey that required a particular response.

Demographic information collected included participants’ age, sex (referring to one’s biological characteristics and/or assignment at birth), gender (referring to one’s gender identity as distinct from sex) and ethnicity. Participants were categorised into three groups – cisgender men, cisgender women and gender-diverse people (encompassing the spectrum of nonbinary and transgender identities) – to examine differences associated with sex assigned at birth and gender identity. Henceforth, we refer to this variable as ‘gender’ though, here, we also examine ‘sex differences’ by comparing cisgender men and women. Cisgender participants encompassed 87% of the overall sample, and of the 13% of gender-diverse participants, 63.4% identified as nonbinary (see Table S3 in Supplemental Materials). The study included five intersex participants, who reported their gender identities to be man (*n* = 1), woman (*n* = 1) and gender diverse (*n* = 3). The overall sample was predominantly White (81%). A breakdown of participant ethnicity is found in Table S4 in Supplemental Materials.

### Materials

#### The Comprehensive Autistic Trait Inventory

The CATI is a self-report questionnaire designed to quantify traits and characteristics qualitatively like those shown in autistic people (English et al., 2021; www.cati-autism.com). The CATI contains 42 items spanning six subscales, each containing seven items. These subscales, while not exhaustive of autistic characteristics, are intended to represent those most readily quantified across the general population and include:

- *Social Interactions* – examining desire for, and self-appraisal in, social interactions.
- *Communication* – examining the use and understanding of non-verbal communicative behaviours.
- *Social Camouflage* – relating to masking and compensatory behaviours used to fit in or appear non-autistic.
- *Self-Regulatory Behaviours* (previously *Repetitive Behaviours*) – describing (typically repetitive) actions and physical behaviours that can help alleviate stress and anxiety.
- *Cognitive Flexibility* (previously *Cognitive Rigidity*) – relating to adaptability to changes, insistence on sameness, and flexibility around routines.
- *Sensory Sensitivity* – describing oversensitivity (or hypersensitivity) to external stimuli across several sensory modalities.^2^

CATI items are presented in the form of statements to which respondents report their level of agreement on a 5-point Likert-type scale containing the options ‘strongly disagree’, ‘somewhat disagree’, ‘neither agree nor disagree’, ‘somewhat agree’ and ‘strongly agree’. Responses are scored on a 1–5 scale (with several reverse-keyed items), which can be aggregated to form a total-scale score (range 42–210 and subscale scores (range, 7–35)), with higher scores indicative of a greater endorsement of autistic traits.

### Community involvement

Early in the development of the CATI, six autistic adults were engaged (by M.C.W.E. and M.T.M.) to establish the content of the CATI, including which trait dimensions to focus on and the types of items that would respectfully represent each dimension. This process provided valuable input concerning survey items piloted in the initial assessments and helped justify the inclusion of a ‘social camouflage’ dimension. Yet, it was the researchers who ultimately decided on the naming of the final six subscales.

For the current study, an autistic research team member recruited a working group of six autistic adults to discuss subscale labels and provide alternatives as necessary. Group members aimed to ensure the labels were respectful and inclusive, and accurately represented the items in each subscale. The autistic working group endorsed the *Social Interactions*, *Communication*, *Social Camouflaging* and *Sensory Sensitivity* labels, but recommended that *Cognitive Rigidity* and *Repetitive Behaviours* subscales be renamed *Cognitive Flexibility* and *Self-Regulatory Behaviours* to avoid negative connotations of language and, for the latter, to represent the purpose of such behaviours better. These changes are reflected throughout this report to encourage users of the CATI to employ the modified nomenclature. An updated version of the CATI with these changes is available in the Supplemental Materials.

### Statistical analyses

Analyses were performed using R v4.2.0 (R Core Team, 2022) and *RStudio* v2023.06.1 + 524 (Posit Team, 2023) primarily using the *lavaan* v0.6-19 (Rosseel, 2012), *psych* v2.4.12 (Revelle, 2024), *semTools* v0.5-6 (Jorgensen et al., 2022), *GPArotation* v2024.3-1 (Bernaards & Jennrich, 2005), *pROC* v1.18.5 (Robin et al., 2011) packages and Jamovi v2.6.24 (The jamovi project, 2024).

#### Primary analyses

The initial set of analyses (1) tested the psychometric properties of the CATI across three groups – non-autistic, formally diagnosed autistic and self-identifying autistic people – and (2) determined whether formally diagnosed and self-identifying autistic people significantly differed, to assess whether combining these groups for subsequent analyses would be appropriate. These analyses were performed on the overall sample, separately on each of the three participant groups (Non-autistic, Autistic _DX_ and Autistic _SELF_), and on the combined autistic groups.

**Figure 1.**
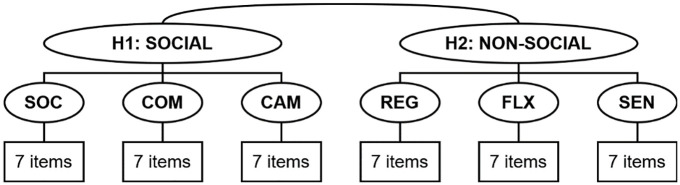
Factor structure of the CATI examined in the present study.

Next, CFAs were performed to test the fit of the CATI factor structure. The original analyses of the CATI conducted on a sample of primarily non-autistic participants (English et al., 2021) indicated support for a bifactor structure that included the six subscales previously described and two additional factors: a social factor comprised of items on the *Social Interactions*, *Communication* and *Social Camouflaging* subscales, and a non-social factor consisting of items on the *Self-Regulatory Behaviours*, *Cognitive Flexibility* and *Sensory Sensitivity* subscales. For the current study, we examine an alternative, hierarchical factor model (see Figure 1) similar to the previously described bifactor model but with the broader social and non-social factors as hierarchical factors directly defined by the previously associated subscales. The hierarchical model was chosen as it lends itself better to the retention of specific factors, or subscales, which can be used for greater specificity by researchers and clinicians. Such specificity may also be key to distinguishing autism from other overlapping conditions, though this challenge is beyond the scope of the current article. Furthermore, it is difficult to say that the bifactor model in English et al. (2021) was definitively *better* than the hierarchical equivalent, given bifactor models are relatively less constrained and generally allow for greater fit than a hierarchical counterpart (Markon, 2019; McFarland, 2016).

CFA was conducted (using *lavaan*) with polychoric correlations and weighted least squares estimation. Model fit was assessed using the comparative fit index (CFI), Tucker–Lewis Index (TLI), root mean square error of approximation (RMSEA) and standardised root mean square residual (SRMR). CFI and TLI >0.90 are typically considered ‘fair’ when used in conjunction with other criteria, and >0.95 ideal, while RMSEA and SRMR are expected to be <0.06 and <0.08, respectively (Hu & Bentler, 1999). For completeness, the overly sensitive chi-square test was also included.

These tests were followed by tests of measurement invariance (using *semTools*) to determine if the factor structure meaningfully differed between the three participant groups (Non-autistic, Autistic _DX_ and Autistic _SELF_) as this would preclude direct comparison of subscale scores. Establishing measurement invariance ensures that CATI scores can be interpreted similarly regardless of group differences (i.e. between different autism or gender groups). This process involves testing a sequence of models with increasingly restrictive parameters and examining whether fit indices substantially change between the following steps: configural invariance (testing the same factor structure across groups), metric invariance (comparing factor loadings) and scalar invariance (examining item intercepts). Changes between models of CFI > 0.01 or RMSEA > 0.015 would indicate a potential violation of the invariance assumption (Chen, 2007; Cheung & Rensvold, 2002).

For all CFAs and tests of measurement invariance, a ‘method’ factor consisting of the reverse-keyed items was included in the model to help account for variance associated with the valence of item phrasing, but the associated results are not reported for the factor.

Correlation and reliability analyses were performed (using *psych*) to observe the strength of associations between and within the CATI subscales. Once the validity of the factor structure and reliability of the CATI were established, we compared total-scale and subscale scores between the three participant groups. Predictive ability of the CATI to classify correctly autistic and non-autistic participants was assessed using binomial logistic regression (using *Jamovi*). Finally, we identified an optimal threshold for discriminating autistic and non-autistic participants by finding the total-scale score that maximised the combined classification accuracy of both autistic (i.e. sensitivity) and non-autistic (i.e. specificity) participants (using *pROC*).

#### Secondary analyses

Additional analyses explored whether gender impacted the psychometric properties of the CATI and investigated differences in autistic trait profiles as a function of this demographic variable. To maximise statistical power, we combined the two autistic groups into a single group (Autistic _ALL_), deemed justifiable following the results of the primary analyses. Otherwise, analyses exploring gender differences followed the methodology of the primary analyses.

## Results

### Re-assessing the CATI using larger clinical samples

#### Verifying the factor structure using confirmatory factor analysis

CFA was performed to examine the fit of the hierarchical factor model defined earlier. The fit indices for the total sample and subsamples (see Table 2) generally exceeded the ‘fair’ fit criteria and surpassed the ‘good’ fit criteria in many cases. Thus, the hierarchical model appears appropriate for each participant subsample.

**Table 2.** Incremental and absolute model fit indices assessing the structure of the hierarchical model of the CATI.

Subsample	χ^2a^	CFI	TLI	SRMR	RMSEA [90% CI]
All participants	8434	0.95	0.94	0.05	0.06 [0.06–0.06]
Non-autistic	5726	0.93	0.93	0.06	0.06 [0.06–0.07]
Autistic _ALL_	5260	0.91	0.90	0.07	0.06 [0.06–0.06]
Autistic _DX_	3601	0.90	0.89	0.07	0.06 [0.06–0.06]
Autistic _SELF_	2481	0.93	0.92	0.07	0.05 [0.05–0.05]
Cisgender man	5023	0.93	0.93	0.06	0.06 [0.06–0.07]
Cisgender woman	3256	0.96	0.96	0.05	0.06 [0.06–0.06]
Gender diverse	1759	0.92	0.92	0.07	0.06 [0.05–0.06]

Acceptable fit criteria was CFI and TLI > 0.90; RMSEA (<0.06) and SRMR (<0.08) (Hu & Bentler, 1999).

a All chi-square tests *df* = 807, *p* < 0.001.

Next, we conducted a multi-group factorial analysis to determine whether the model fit was comparable between the participant groups. We first tested for invariance between the two groups of autistic participants, Autistic _DX_ and Autistic _SELF_ (Comparison 1 in Table 3). The finding of both scalar (‘strong’) and residual (‘strict’) invariance indicates that the hierarchical, six-factor structure showed comparable fit for the two autistic groups and that their scores can be compared directly and meaningfully (Wu et al., 2007). Given the invariance between Autistic _DX_ and Autistic _SELF_ groups, we combined them into a single, larger group (Autistic _ALL_) to test for invariance with non-autistic participants (Comparison 2 in Table 3). As with the previous test, both scalar (‘strong’) and residual (‘strict’) invariance was indicated, meaning the hierarchical factor structure was comparable in fit for the autistic and non-autistic participants.

**Table 3.** Results of multiple-group factorial analyses assessing measurement invariance of the CATI as a function of participant subsample, comparing (1) formally diagnosed autistic and self-identifying autistic participants, (2) non-autistic and autistic participants (combined formally diagnosed and self-identifying), and (3) cisgender men, cisgender women, and gender-diverse participants.

Model	χ^2^	*df*	CFI	RMSEA (90% CI)	Δχ^2^	Δ*df*	ΔCFI	ΔRMSEA	Decision
**Comparison 1: Autistic _DX_ versus Autistic _SELF_**									
Configural invariance	6558	1612	0.964	0.068 (0.066–0.070)	-	-	-	-	Accept
Metric (weak) invariance	6688	1732	0.964	0.066 (0.064–0.068)	130	120	0.000	-0.002	Accept
Scalar (strong) invariance	6787	1774	0.963	0.065 (0.064–0.067)	99	42	0.001	-0.001	Accept
Residual (strict) invariance	7074	1816	0.961	0.066 (0.065–0.068)	287	42	0.002	0.001	Accept
**Comparison 2: Non-autistic versus Autistic _ALL_**									
Configural invariance	12325	1612	0.971	0.072 (0.070–0.073)	-	-	-	-	Accept
Metric (weak) invariance	12968	1732	0.969	0.071 (0.070–0.072)	643	120	0.002	-0.001	Accept
Scalar (strong) invariance	13261	1774	0.968	0.071 (0.069–0.072)	293	42	0.001	0.000	Accept
Residual (strict) invariance	14301	1816	0.966	0.073 (0.072–0.074)	1039	42	0.002	0.002	Accept
**Comparison 3: Cisgender Man versus Cisgender Woman versus Gender Diverse**									
Configural invariance	9484	1612	0.988	0.066 (0.064–0.067)	-	-	-	-	Accept
Metric (weak) invariance	9635	1732	0.988	0.064 (0.062–0.065)	151	120	0.000	-0.002	Accept
Scalar (strong) invariance	10112	1774	0.987	0.064 (0.063–0.066)	477	42	0.001	0.000	Accept
Residual (strict) invariance	10376	1816	0.987	0.065 (0.063–0.066)	264	42	0.000	0.001	Accept

ΔCFI > 0.01 and ΔRMSEA > 0.015 indicate a violation of the invariance assumption. CFI: comparative fit index; RMSEA: root mean square error of approximation (Chen, 2007; Cheung & Rensvold, 2002).

#### Correlations between subscales

Next, we performed a series of correlation analyses to assess the strength of the relationships between the CATI subscale scores – for the overall sample, the two autistic participant groups, separately and combined, and the non-autistic participant group – to determine whether variations existed as a function of autism status or subgroup. Across all groups, correlations between the six subscales were significant and positive (*r* range = 0.12–0.64, *r* mean (total sample) = 0.35, largest *p* = 0.01; full details in Supplemental Table S5).

#### Internal consistency reliability

Internal consistency reliability of the CATI and its subscales calculated using both Cronbach’s *alpha* and McDonald’s *omega* are summarised in Table 4 for the total sample and separate participant groups. Cronbach’s *alpha* and McDonald’s *omega* exceeded generally accepted thresholds for all groups across the total scale, higher-order subscales and specific subscales. *Omega hierarchical*, which indicates the reliability of an overall general factor, was relatively lower, suggesting that the total score is not strongly representative of a single underlying autistic trait dimension. As the *omega hierarchical* is based on inter-subscale correlations (as opposed to inter-item correlations), these weaker reliability estimates reflect the relatively modest correlations between individual subscales (see Supplemental Table S5). Relatively weaker omega hierarchical is also not surprising given diagnostic manuals for autism refer to multiple dimensions (e.g. *Diagnostic and Statistical Manual of Mental Disorders*, 5th ed., text rev., DSM-5-TR; APA, 2022), and users of the CATI would likely benefit in considering the subscale scores alongside any interpretation of the total-scale score.

**Table 4.** Internal consistency reliability (omega hierarchical) for the total sample and separately for diagnostic status and gender subgroups.

Subsample	Cronbach alpha			McDonald’s omega			Omega hierarchical
	Total scale	Higher order subscales	Specific subscales	Total scale	Higher order subscales	Specific subscales	
All participants	0.96	0.93–0.94	0.85–0.94	0.97	0.94–0.95	0.89–0.95	0.68
Non-autistic	0.93	0.89–0.91	0.82–0.94	0.95	0.92–0.94	0.86–0.95	0.54
Autistic _ALL_	0.92	0.87–0.89	0.80–0.89	0.93	0.91–0.91	0.86–0.92	0.49
Autistic _DX_	0.93	0.88–0.90	0.81–0.90	0.94	0.91–0.92	0.86–0.93	0.77
Autistic _SELF_	0.90	0.86–0.87	0.78–0.88	0.92	0.90–0.91	0.85–0.91	0.36
Cisgender man	0.95	0.92–0.92	0.84–0.94	0.96	0.93–0.95	0.88–0.96	0.61
Cisgender woman	0.96	0.94–0.95	0.86–0.94	0.97	0.95–0.96	0.89–0.96	0.73
Gender diverse	0.94	0.91–0.91	0.80–0.91	0.95	0.93–0.94	0.86–0.94	0.53

For Cronbach’s alpha and McDonald’s omega, values > 0.80 are expected for research, but >0.70 is also generally acceptable (Taber, 2018; Tavakol & Dennick, 2011). Values >0.90 may indicate redundancy between items (Streiner, 2003).

#### Score distributions and diagnostic-status group comparisons

**Figure 2.**
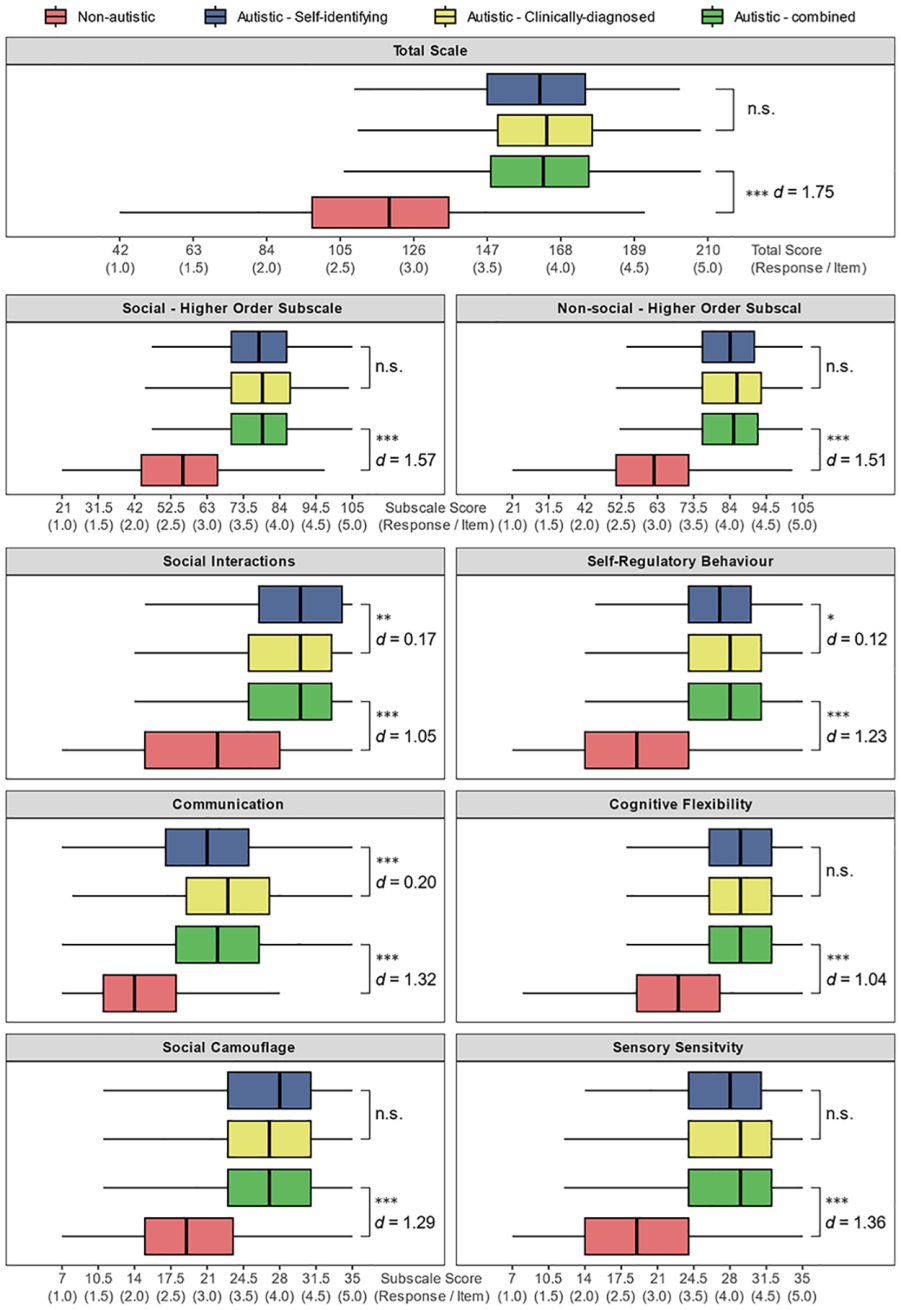
Box-and-whisker summaries of CATI total scores and subscale scores divided by participant group. The mid-line represents the group median, and the whiskers extend to the furthest score within the inter-quartile range multiplied by 1.5. Independent samples *t*-tests summarise comparisons between the two autistic groups (Autistic _DX_ vs Autistic _SELF_) and between the non-autistic group and the combined autistic group. n.s. = non-significant. *p < 0.05. **p < 0.01. ***p < 0.001.

Box-and-whisker plots for the CATI total-scale and subscale scores separated by diagnostic-status group revealed differences in total scores and subscale scores between non-autistic and autistic groups (Figure 2; see Table S6 in Supplemental Materials for additional statistics). While independent samples *t*-tests demonstrated that CATI total-scale and hierarchical subscale scores did not differ significantly between Autistic _DX_ and Autistic _SELF_ participants, differences were apparent on some CATI subscales. Specifically, the Autistic _SELF_ group had higher scores on the *Social Interactions* subscale than the Autistic _DX_ group. At the same time, the reverse was found for the *Communication* and *Self-Regulatory Behaviours* subscales. However, the effect sizes for these three differences were relatively small (all *d*s ⩽ 0.20, see Figure 2).

These analyses were followed with a similar set of *t*-tests comparing non-autistic participants and the combined autistic groups (Autistic _ALL_). As expected, all tests were significant and showed large effect sizes (all *p*s < 0.001, all *d*s > 1.04; see Figure 2). Autistic participants scored substantially higher on the CATI total-scale score and all subscales compared to non-autistic participants.

#### Assessing the predictive capacity to classify autism using logistic regression analyses

Next, criterion-related validity was assessed by performing logistic regression analyses that determined how well total-scale CATI scores distinguished between autistic and non-autistic participants. We selectively focused on non-autistic and autistic participants with a formal diagnosis participants to perform a binary logistic regression (0 = non-autistic, 1 = autistic). The results of this analysis (see Table 5) indicate that the total-scale score was a good predictor of autism status, with Nagelkerke’s pseudo *R*^2^ suggesting that this single variable accounted for 54% of the total variance in autism status. Identical logistic regressions were conducted using the two higher-order subscales and six specific subscales as predictors (see **Table S7** and **S8** in the Supplemental Material). Small incremental improvements were observed, particularly when using the six subscales (i.e. 54% vs 57% variance explained for the overall sample using total-scale and specific subscale scores, respectively). However, the small benefit in increased classification performance must be weighed against the significant additional complexity introduced by multiple predictors.

**Table 5.** Model summaries, including standardised coefficients, of a logistic regression analysis predicting autism status (i.e. Autistic _**DX**_ vs non-Autistic) using the CATI total scale score for the total sample and separately for gender identity subgroups.

	β	Wald test		Odds ratio	95% CI for odds ratio	
		Wald	*p*		Lower	Upper
**Total sample**						
***df*** **=** **1, χ**^2^ **=** **1018, *p*** **<** **0.001, Nagelkerke’s pseudo *R***^2^ **=** **0.542, AUC** **=** **0.888**						
(Intercept)	-9.791	523	<0.001	0.000	0.000	0.000
*Total Scale Score*	0.066	507	<0.001	1.069	1.068	1.074
**Cisgender man**						
***df*** **=** **1, χ**^2^ **=** **314, *p*** **<** **0.001, Nagelkerke’s pseudo *R***^2^ **=** **0.390, AUC** **=** **0.830**						
(Intercept)	-7.739	218	<0.001	0.001	0.000	0.001
*Total Scale Score*	0.051	197	<0.001	1.053	1.045	1.060
**Cisgender woman**						
***df*** **=** **1, χ**^2^ **=** **556, *p*** **<** **0.001, Nagelkerke’s pseudo *R***^2^ **=** **0.660, AUC** **=** **0.929**						
(Intercept)	-12.171	223	<0.001	0.000	0.000	0.000
*Total Scale Score*	0.082	219	<0.001	1.085	1.073	1.097
**Gender diverse**						
***df*** **=** **1, χ**^2^ **=** **113, *p*** **<** **0.001, Nagelkerke’s pseudo *R***^2^ **=** **0.577, AUC** **=** **0.905**						
(Intercept)	-13.050	44	<0.001	0.000	0.000	0.000
*Total Scale Score*	0.088	48	<0.001	1.092	1.065	1.120

#### Sensitivity, specificity, and a useful ‘cut-off’ for classifying autism

While the CATI was initially designed for assessing autistic traits in non-autistic people, it potentially has additional utility as a screener for identifying possible autism in research, clinical, and personal settings. To support this utility, we identified the optimal threshold score that best discriminated between autistic participants who reported having a formal diagnosis (Autistic _DX_) and non-autistic participants. A score of 147.5 was associated with the maximum possible combined specificity (87.41%) and sensitivity (77.20%) (see Table 6 for confidence intervals). A 95% confidence interval (10,000 ordinary bootstrapped samples) of 140.5–150.5 around the threshold was calculated, indicating the greatest classification uncertainty score range.

**Table 6.** Classification indices and 95% bootstrapped confidence intervals (10,000 samples), including ‘best’ total-scale score thresholds for discriminating non-autistic and diagnosed autistic participants across the total sample and separately by gender.

	Autism threshold	Autistic _DX_ (sensitivity)	Non-autistic (specificity)
All participants	147.5 [140.5–150.5]	77.20 [73.27–82.63]	87.41 [81.63–90.46]
Cisgender man^a^	147.5 [134.5–151.5]	66.03 [59.94–80.45]	86.36 [72.09–91.01]
Cisgender woman^a^	145.5 [136.5–149.5]	85.52 [81.82–92.59]	89.93 [82.91–92.81]
Gender diverse^a^	150.5 [149.5–163.5]	88.98 [68.50–93.70]	76.92 [73.08–96.15]

a Note the confidence interval for each gender-specific threshold overlaps with the threshold confidence interval for all participants, indicating no meaningful advantage in using a gender-specific threshold currently.

### Assessing the interaction between gender and autism on CATI scores

Having identified satisfactory psychometric properties for the CATI when considering the overall sample, we next examined the psychometric properties in separate gender groups. We conducted a series of analyses like the first but employing gender identity (cisgender man, cisgender woman and gender diverse) as an additional grouping factor, with the former two categories representing sex-assigned-at-birth differences and gender-diverse participants also represented. Our analysis revealed little meaningful distinction between formally diagnosed and self-identifying autistic participants; we, therefore, combined these groups to enable a more robust examination of potential sex and gender differences.

#### Verifying the factor structure using confirmatory factor analysis

As a first step, using separate confirmatory CFAs, we tested the goodness of fit for the hierarchical model of the CATI for each of the three gender groups, collapsed across autism group. The results (summarised in Table 2) indicate that all groups showed levels of fit at least ‘fair’ and often ‘good’ without substantial variability in fit indices between the groups.

#### Measurement invariance

Having established that model fits were satisfactory for each gender group independently, we then directly compared model fit for the hierarchical model as a function of gender (cisgender man vs cisgender woman vs gender diverse), collapsed across autism group. As before, a series of factor models were tested with greater restrictions on model parameters implemented in subsequent models and with scalar (strong) invariance typically required to make meaningful comparisons of factor scores between different groups. The data (summarised in Table 3) provide evidence for measurement invariance, indicating that the scale structure does not differ substantially as a function of gender and autism status and that, therefore, total-scale and subscale scores between these groups may be compared directly and meaningfully.

#### Correlations between subscales

Having established measurement invariance for autism status and gender, we then explored correlations between subscales for each gender. Significant positive correlations between all six subscales were evident across all three gender groups (*r* range = 0.23–0.66, *r* mean (all gender groups) = 0.51, largest *p* < 0.001; full details in **Table S9** in Supplemental Materials).

#### Internal consistency reliability

Internal consistency was assessed for each comparison group using a set of reliability analyses identical to those used in the first section (see Table 4). As before, Cronbach’s *alpha* and McDonald’s *omega* values were generally acceptable for the total scale and subscales. Again, *omega hierarchical* was relatively lower, suggesting caution when solely interpreting the total-scale alone and encouraging the use of the subscales.

#### Score distributions and group comparisons

**Figure 3.**
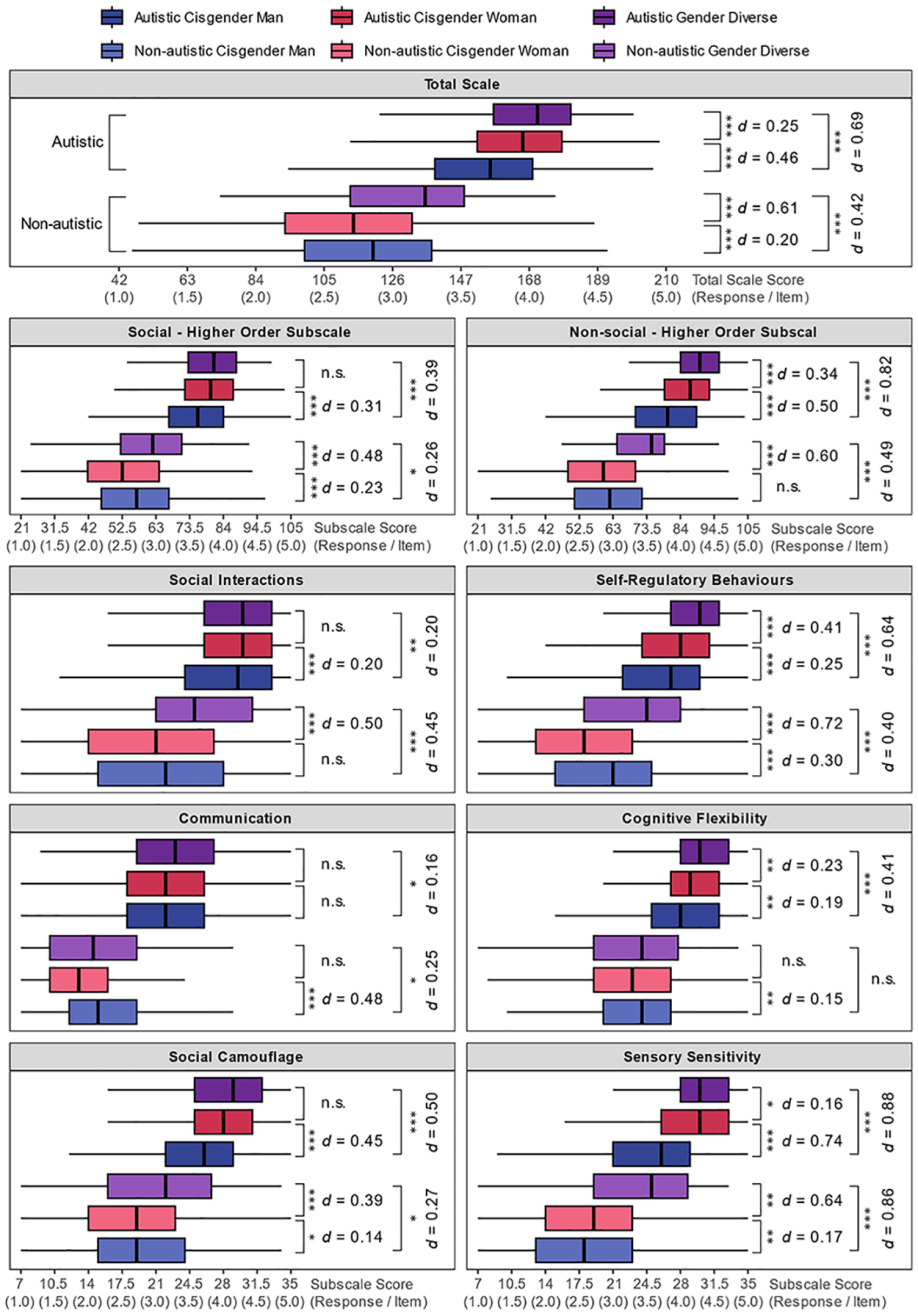
Box-and-whisker summaries of CATI total and subscale scores divided by autism group and gender. The mid-line represents the group median, the box represents the inter-quartile range, and the whiskers extend to the furthest score within the interquartile range multiplied by 1.5. Independent samples *t*-tests summarise specific gender comparisons within the autism groups. n.s. = non-significant. *p < 0.05. **p < 0.01. ***p < 0.001.

To illustrate differences in the distribution of total-scale and subscale scores as a function of both autism status and gender, box-and-whisker plots were generated and annotated with Tukey’s Honestly Significant Difference (HSD) test outcomes (see Figure 3; see **Table S10** in Supplemental Materials for additional details). Given that previous analyses for the overall sample established large differences between scores for autistic and non-autistic participants, the current analyses focused on gender differences for autistic and non-autistic participants separately. In addition, given the high levels of similarity between diagnosed and self-identifying autistic participants, the two groups were collapsed together for these gender analyses. Among autistic people, gender-diverse respondents showed the highest CATI total scores, followed by cisgender women and then cisgender men. Among non-autistic participants, gender-diverse participants again showed the highest scores, but were instead followed by cisgender men, with cisgender women showing the lowest scores.

With respect to subscales, a complex pattern of gender differences was evident across the six autistic trait dimensions. Among non-autistic participants, the gender-diverse participants showed substantially higher scores for *Social Interactions*, *Social Camouflage*, *Self-Regulatory Behaviour* and *Sensory Sensitivity* than cisgender men and cisgender women. *Communication* scores were lowest for cisgender women and comparable between cisgender men and gender-diverse participants. In contrast, *Cognitive Flexibility* scores were broadly similar across gender categories (largest *d* = 0.15).

Among autistic participants, cisgender women and gender-diverse participants showed higher *Social Camouflage* and *Sensory Sensitivity* scores than cisgender men, while gender-diverse participants showed the highest scores for *Self-Regulatory Behaviour*. In contrast, *Social Interaction* and *Communication* scores were relatively similar across gender groups (largest *d* = 0.21). *Cognitive Flexibility* scores increased from cisgender men to cisgender women and then to gender-diverse participants. Interestingly, the largest differences were observed on the *Sensory Sensitivity* subscale among autistic participants, where cisgender women and gender-diverse participants showed much higher scores than cisgender men (both *d*s > 0.78).

#### Gender differences in performance of classifying autism

Next, using binomial logistic regression analyses, we assessed the capacity of the CATI total-scale scores to discriminate between the Autistic _ALL_ and non-autistic participants in each gender group (see Table 5). Nagelkerke’s pseudo-*R*^2^ was largest for cisgender women, followed by gender-diverse participants, and smallest for cisgender men, with the models accounting for 66%, 58% and 39% of the total variance, respectively.

Finally, we calculated gender-specific thresholds for identifying autism using the same calculation procedure used for the overall sample. Total-scale scores of 147.5, 145.5 and 150.5 maximised the discriminability of autistic and non-autistic participants for cisgender men, cisgender women and gender-diverse participants, respectively. However, as all the 95% confidence intervals around these thresholds (see Table 6) overlapped with each other and the gender-nonspecific threshold of 147.5 previously reported, the current data suggests there is no meaningful advantage in using gender-specific autism thresholds. CATI users are recommended to use the general threshold of 147.5, noting the 95% confidence interval of 140.5–150.5 around the threshold.

## Discussion

The present study significantly extends the original psychometric analysis of the CATI (English et al., 2021) by providing the first psychometric evaluation that includes a large sample of autistic and non-autistic people. The psychometric performance of the CATI in both diagnosed and self-identifying autistic participants was comparable and largely distinguishes autistic and non-autistic groups. Furthermore, the structure and reliability of the CATI was found to be comparable across different genders, including gender-diverse participants, suggesting that the CATI may be appropriate across a broad demographic range. We now examine the impact of our findings in the broader autism context.

### General psychometric performance

Our analyses permitted a much deeper psychometric examination of the CATI than English et al. (2021) achieved, and the results are encouraging in terms of a clear identification of phenotypic differences for autistic and non-autistic populations. A CFA for the total sample produced fit indices supporting a hierarchical factor structure of the CATI with two broader higher level factors, each subsuming three lower level factors. Critically, this factor structure was supported across all three autism groups – formally diagnosed autistic adults, self-identifying autistic adults and non-autistic adults from the general population. This psychometric similarity was further supported by the observed measurement invariance between the autistic and non-autistic groups; thus, there were no meaningful differences in how the different groups completed the CATI, and their total and subscale scores could be meaningfully compared. Reliability analyses were similarly supportive of this perspective, with total-scale and subscale Cronbach’s *alpha* and McDonald’s omega values exceeding the minimum of 0.70 recommended for research purposes for the overall sample and for each individual group of autistic and non-autistic participants (Taber, 2018; Tavakol & Dennick, 2011). That said, *omega hierarchical* values were notably lower, suggesting caution when using the total-scale score alone to classify autism. This outcome is not entirely surprising – diagnostic manuals, such as the DSM-5-TR (APA, 2022), contain clinical descriptions of autism with separate social and non-social domains, and thus users of autistic trait scales and screeners should examine total-scale scores along with subscale scores in their classification making decisions.

The present study re-evaluated the cut-off for classifying autistic and non-autistic people, taking advantage of the larger sample. A total-scale score of 147.5 was found to best discriminate between diagnosed autistic and non-autistic people, achieving classification accuracy of 77% and 87%, respectively, for these two groups. These values should be particularly useful for clinicians wishing to introduce the CATI into screening procedures. The classification rates are respectable, considering the scale was not developed first and foremost as a screening tool and given the continuum-like nature of autism across the general population. The total-scale threshold of 147.5 and above is higher than that calculated in the original study (134 and above; English et al., 2021), possibly due to using a larger, more representative autistic sample in the current study. The calculation of a confidence interval for the total-score threshold (i.e. 140.5–150.5) is intended to emphasise particular caution in interpreting individual scores within this range.

### Similarity of formally diagnosed and self-identifying autistic participants

A notable outcome of our study is the high concordance between formally diagnosed and self-identifying autistic participants across all analyses and the finding of measurement invariance for the factor structure across the two groups. While the two groups differed significantly on several subscales, the effects were relatively small (i.e. d ⩽ .20; Cohen, 2013). Furthermore, the differences were not always in the suspected direction (i.e. that self-identifying autistic people would have lower scores), suggesting that self-identifying autistic people may not simply be displaying ‘milder’ traits compared to those who have received a formal diagnosis. Comparable similarities have been noted on other measures – for example, Sturm et al. (2024) reported striking similar psychometric properties on the RAADS-R between adults with an autism diagnosis, and adults self-identifying as autistic without a diagnosis.

One interpretation of the high level of similarly between self-identifying and clinically diagnosed autistic participants in the present study is that the two groups *are* similar except for the path towards autistic identity. If true, this would tentatively support the inclusion of self-identifying autistic people in autism research, which would yield several benefits, such as increasing the potential pool of ‘autistic’ participants available for research studies and make autistic samples more representative by including people who lack ‘official’ diagnoses for various reasons (e.g. gender biases, limited access to diagnostic services and prohibitive costs of diagnostic evaluations; (Anderson et al., 2018; Botha et al., 2020; Kapp et al., 2013; Lewis, 2017; Lockwood Estrin et al., 2021). However, work using other screening instruments has tended to find low concordance between adults identified as autistic using existing screening instruments and their likelihood of receiving a clinical diagnosis (for review, see Wigham et al., 2019). It is possible that previous screeners simply underperformed in identifying autism, and a sufficiently well-designed screening instrument would not face this issue, but the CATI remains untested in this regard.

To our knowledge, no research has examined what proportion of self-identified autistic adults who seek formal assessment ultimately receive a clinical autism diagnosis. Understanding to what extent clinicians and formal diagnostic methods agree with self-identification is an issue that is well worth investigating further. The formation of an autistic identity, either through self-identification or diagnosis, contributes positively to the mental health and well-being of autistic people (Bervoets & Hens, 2020; Davies et al., 2023; Fletcher-Watson, 2023; Leedham et al., 2019; Lewis, 2016; Lilley et al., 2021), facilitating access to appropriate support that can improve quality of life, and shifting perceptions from negative labelling to a more nuanced understanding of personal identity (Arnold et al., 2020; Cerda et al., 2023; Davies et al., 2023; Huang et al., 2022a, 2022b; Hume & Burgess, 2021; McDonald, 2020; Smith-Young et al., 2020). Establishing reliable and accessible pathways to self-identity and developing ways to validate self-identity, potentially with tools such as the CATI, would magnify the positive outcomes described above.

### Gender differences

The original work developing the CATI included a limited set of analyses comparing cisgender men and women, reporting the crucial finding of measurement invariance and several sex differences between cisgender men and women (English et al., 2021). Here, we extended these analyses by examining the interaction between gender and autism status, as well as considering gender-diverse participants in addition to cisgender men and women. Fit indices calculated from separate CFAs indicated that the hierarchical factor structure of the CATI was supported in cisgender men, cisgender women and – for the first time – in gender-diverse adults. Reliability indices for each gender were similar to that for the overall sample. Together, these findings provide confidence in using the CATI across gender identities.

While the psychometric properties of the CATI across genders were similar, several gender differences in the total-scale and subscale scores were evident, with a relatively complex pattern emerging. Among non-autistic participants, cisgender men had particularly high *Communication* and *Self-Regulatory Behaviour* scores compared to cisgender women. Among autistic participants, cisgender women reported more pronounced autistic traits than cisgender men for the total scale and every subscale except *Communication*. Thus, taking the autistic and non-autistic participants together, cisgender women showed a larger range of scores than cisgender men, consistent with outcomes from the AQ (Baron-Cohen et al., 2001). Finally, gender-diverse participants reported higher scores for the total scale and most of the subscales compared to cisgender men and cisgender women, with these differences observed within both the autistic and non-autistic groups. However, the gender differences were much more pronounced among the non-autistic groups, where the gender-diverse group reported trait levels midway between the levels reported by the cisgender non-autistic groups, and the autistic groups (consistent with Warrier et al., 2020).

One general outcome from our study is that gender differences in traits cannot be assumed to be identical for autistic and non-autistic people, as we report many instances where gender differences were found for one group but not the other, or they presented in opposite directions. For example, while autistic cisgender women showed higher *Self-Regulatory Behaviour* and *Cognitive Flexibility* scores relative to autistic cisgender men, the reverse was true for non-autistic participants (i.e. cisgender women < cisgender men).

Notably, while we found traits associated with social-camouflaging behaviours and sensory sensitivity to be markedly higher in autistic cisgender women compared to autistic cisgender men, these dimensions are often underrepresented (or not represented at all) in older measures of autistic traits (Baron-Cohen et al., 2001; Hurley et al., 2007). This historical absence of the representation of trait dimensions more characteristic of autistic cisgender women compared to autistic cisgender men is likely linked to the traditional concept of autism as a condition predominantly affecting males (Aykan et al., 2020). On one hand, the inclusion of traits such as heightened sensory sensitivity and social camouflage in the CATI may serve to narrow the gender gap in autistic traits. However, the inclusion of these traits may also reduce diagnostic specificity when attempting to discern autism from other conditions given many other neuropsychiatric conditions feature traits such as altered sensory sensitivity or anxiety-linked social masking (Ahmmed & Mukherjee, 2021; Bijlenga et al., 2017; Miller et al., 2021).

Furthermore, the diagnostic process for gender-diverse people or cisgender women can be complicated by additional factors, including stigma or gender-based interpretation biases from referral sources (e.g. family, school, general practitioners) and a greater likelihood of diagnostic overshadowing by other conditions (e.g. other neurodevelopmental disorders, anxiety, depression) or misdiagnoses (e.g. borderline personality disorder or gender dysphoria) (Bysph et al., 2023; Hilton et al., 2022; Lai et al., 2015). The consequence of ‘biased’ assessment criteria is that they likely do not fully represent the breadth of autistic presentations (here, specifically non-male presentations), meaning women were more likely to miss opportunities for diagnosis (potentially accounting for a later average age of diagnosis in women compared to men), and that cisgender women needed ‘more’ traits before a diagnosis was conferred (D’Mello et al., 2022; Glidden et al., 2016; Warrier et al., 2020). The latter contention is supported by the current study (i.e. autistic cisgender women reported higher scores than autistic cisgender men) and in previous reports (e.g. using the AQ – Baron-Cohen et al., 2001). Furthermore, as diagnostic criteria have now broadened to be generally more inclusive, prevalence rates between males and females have become considerably more comparable (Zeidan et al., 2022).

The observation that self-identified gender-diverse participants showed higher scores in several trait domains could reflect the fact that some autistic characteristics may overlap with the characteristics of gender-diverse people. For example, gender-diverse people can show camouflaging to ‘pass’ as a particular gender or increased sensory sensitivities towards aspects of their body that are nonconformant with their sense of gender (Hilton et al., 2022; Kallitsounaki & Williams, 2023). Future assessments might better distinguish between characteristics that are linked to both autism and gender, though it is unlikely that such traits can be perfectly disentangled. Similarly, there is growing recognition that the prevalence of autism is substantially higher among gender-diverse people than among cisgender men and cisgender women (Nobili et al., 2018; Warrier et al., 2020), with a recent meta-analysis estimating rates of autism to be up to 11 times higher for gender-diverse people compared to the general population (Kallitsounaki & Williams, 2023). Given the interaction between gender diversity and autism and that gender-diverse people are estimated to account for 0.1%–2% of the population (Goodman et al., 2019), future research might concentrate on confirming whether autistic trait measures are psychometrically appropriate for gender-diverse people in addition to cisgender men and women.

### Limitations and future research

The current study develops the promising findings of the original description of the CATI (English et al., 2021) and highlights its utility as a validated and up-to-date measure of autistic traits. Nevertheless, several outstanding issues remain to be addressed. First, our sample of diagnosed autistic participants relied on self-reporting of a diagnosis without confirmation by a clinician. We also did not distinguish between diagnostic methods or historical diagnostic categories such as Asperger’s syndrome. Future studies should aim to test the replicability of our findings in a clinically confirmed autistic population.

Further to this point, and as noted above, the real-world application of the CATI as a screening tool should be tested by examining how well the CATI identifies subsequent autism diagnoses in adults undertaking an autism assessment. As outlined in studies that have examined this issue using other screening instruments (for review, see Wigham et al., 2019), the results have tended call into question the accuracy of screening instruments in clinical settings. Until the CATI’s predictive capacity is tested in such applied settings, clinicians should refrain from relying heavily on the results provided by the CATI in their decision-making process. Similarly, future research should examine self-identification accuracy more deeply, particularly the concordance between self-identification and subsequent clinical diagnosis (assuming accurate diagnoses), and involving groups not extensively represented in this study (e.g. multiply marginalised or of minority racial/ethnic backgrounds).

Second, the classification accuracy of the proposed scoring thresholds is intrinsically related to how accurately autism is diagnosed and self-reported in our samples and the extent to which people identifying as non-autistic have been adequately screened. For example, it has been argued that females are historically underdiagnosed (Belcher et al., 2022; Lockwood Estrin et al., 2021) and may require relatively ‘more’ autistic traits than their male counterparts before a positive diagnosis is proffered. As diagnostic practices change over time and autism in cisgender women is better recognised, future studies like the current one may see downward trends in the overall trait levels of autistic cisgender women, with the result of potentially lower thresholds than what we report here. A similar outcome may also be apparent for gender-diverse people, as autism becomes better identified for this group and more nuanced analyses are developed for specific gender identities (e.g. nonbinary, trans, genderfluid). These issues affect all autism screening tools to some extent, given that classification accuracy is intrinsically linked to the accuracy of current diagnostic practices.

It is well established that autism co-occurs with a range of conditions (Mosner et al., 2019), increasing the complexity of diagnosis. We did not distinguish between autism presenting with the many other conditions reported to co-occur with autism (e.g. anxiety disorders, attention-deficit hyperactivity disorder, obsessive-compulsive disorder) (Johnston et al., 2013; Mosner et al., 2019). Future use of the CATI should examine the impact of co-occurring conditions on autistic traits, an issue the present research team is actively working on, in addition to how well the CATI can accurately classify autism in people with common co-occurring conditions in the context of screening.

Finally, future work should examine the outcomes of several other psychometric tests to determine the extent to which the CATI is a useful clinical and research tool. Test–retest reliability should be established to assess the stability of the CATI at different measurement time points. While convergent validity and classification performance of the CATI compared to the AQ (Baron-Cohen et al., 2001) and BAPQ (Hurley et al., 2007) was examined in the original study, similar tests should be performed against more contemporary measures such as the SRS-A (Aldridge et al., 2012) and RAADS-R (Ritvo et al., 2011). Comparisons should also be made against the CATI-R (Hechler et al., 2025), a heavily modified version of the original CATI that aimed to improve further the clarity and respectfulness of language (and published late in the revision process of the present study). While on the surface, the alternatively worded and edited CATI-R may appear interchangeable with the original, it is highly likely that the underlying psychometric properties differ and may not be directly applicable to each other (e.g., the threshold for identifying autism calculated in the present study may not be valid in the CATI-R).

## Conclusion

The concept of autistic trait dimensions that can be assessed in general and clinical populations remains relevant to autism researchers, clinicians making diagnostic decisions and members of the public seeking to understand autism and, potentially, themselves better. To that end, the available tools for measuring autistic traits must reflect the current understanding of autism in an accessible and respectful manner. The CATI is one such measure. Our findings build upon the strong psychometric results reported for the CATI (English et al., 2021) by demonstrating, in a large sample of diagnosed and self-identifying autistic participants, that the CATI and its six subscales continue to demonstrate a consistent factor structure and high levels of reliability. Furthermore, measurement invariance was established as a function of autism diagnostic status and gender, meaning that CATI scores can be meaningfully interpreted and compared between the different demographic groups, crucially including those of gender-diverse backgrounds. These findings hold promise for researchers and clinicians seeking alternatives to older general measures of autistic traits that may not reflect the currently known breadth of autistic traits. However, we also stress that further psychometric study is encouraged to assess the usefulness of the CATI in a clinical context.

## Supplemental Material

sj-docx-1-aut-10.1177_13623613251347740 – Supplemental material for Psychometric evaluation of the Comprehensive Autistic Trait Inventory in autistic and non-autistic adults Supplemental material, sj-docx-1-aut-10.1177_13623613251347740 for Psychometric evaluation of the Comprehensive Autistic Trait Inventory in autistic and non-autistic adults by Michael CW English, Rebecca E Poulsen, Murray T Maybery, David McAlpine, Paul F Sowman and Elizabeth Pellicano in Autism

sj-zip-1-aut-10.1177_13623613251347740 – Supplemental material for Psychometric evaluation of the Comprehensive Autistic Trait Inventory in autistic and non-autistic adults Supplemental material, sj-zip-1-aut-10.1177_13623613251347740 for Psychometric evaluation of the Comprehensive Autistic Trait Inventory in autistic and non-autistic adults by Michael CW English, Rebecca E Poulsen, Murray T Maybery, David McAlpine, Paul F Sowman and Elizabeth Pellicano in Autism

## Notes

1. In Samples 3 and 4, four items in the *Sensory Sensitivity* subscale had words adjusted to improve comprehension following recommendations from an autistic advisory group. Measurement invariance was found for the original and revised wording (see Table S2 in Supplemental Material), suggesting outcomes using the differing wording are statistically comparable. We encourage using the updated phrasing for future use of the Comprehensive Autistic Trait Inventory (CATI) and have updated the online materials to reflect the changes. The edited items are as follows with the original text in parenthesis. Item 4: I am (**over**-) **very** sensitive to bright lighting; Item 18: I am (**over**-) **very** sensitive to touch; Item 24: I am (**over**-) **very** sensitive to particular tastes (e.g. salty, sour, spicy, or sweet); Item 36: I react (**poorly**) **strongly** to unexpected loud noises.

2. For brevity, the CATI does not include hyposensitivity items. However, studies using the Glasgow Sensory Questionnaire (Robertson & Simmons, 2013) have found positive correlations between hyper- and hyposensitivity subscale scores in autistic adults and therefore limited evidence of statistical separation of the two item categories (Kuiper et al., 2019). In addition, autistic versus non-autistic differences have been reported in hypersensitivity, but not hyposensitivity, using the Sensory Perception Quotient (Taylor et al., 2020).

## References

[bibr1-13623613251347740] Adamou M. Jones S. L. Wetherhill S. (2021). AAA screening in adults with ASD: A retrospective cohort study. Advances in Autism, 8(3), 232–242. 10.1108/AIA-10-2020-0059

[bibr2-13623613251347740] Adkin T. (2023, May 8). Mask on, Mask off: How the common understanding of Autistic masking is creating another mask. Emergent Divergence. https://emergentdivergence.com/2023/05/08/mask-on-mask-off-how-the-common-understanding-of-autistic-masking-is-creating-another-mask/

[bibr3-13623613251347740] Agelink van Rentergem J. A. Lever A. G. Geurts H. M . (2019). Negatively phrased items of the Autism Spectrum Quotient function differently for groups with and without autism. Autism, 23(7), 1752–1764. 10.1177/1362361319828361 30818972 PMC6728748

[bibr4-13623613251347740] Ahmmed A. U. Mukherjee D. (2021). Auditory processing and non-auditory factors associated with hyperacusis in children with auditory processing disorder (APD). Hearing Balance and Communication, 19(1), 1–12. 10.1080/21695717.2020.1727216

[bibr5-13623613251347740] Aldridge F. J. Gibbs V. M. Schmidhofer K. Williams M. (2012). Investigating the clinical usefulness of the Social Responsiveness Scale (SRS) in a tertiary level, autism spectrum disorder specific assessment clinic. Journal of Autism and Developmental Disorders, 42(2), 294–300. 10.1007/s10803-011-1242-9 21516433

[bibr6-13623613251347740] American Psychiatric Association. (2022). Diagnostic and statistical manual of mental disorders (5th ed., text rev.). American Psychiatric Association.

[bibr7-13623613251347740] Anderson C. Lupfer A. Shattuck P. T. (2018). Barriers to receipt of services for young adults with autism. Pediatrics, 141(Suppl. 4), S300–S305. 10.1542/peds.2016-4300G 29610411

[bibr8-13623613251347740] Arnold S. R. C. Huang Y. Hwang Y. I. Richdale A. L. Trollor J. N. Lawson L. P. (2020). ‘The single most important thing that has happened to me in my life’: Development of the Impact of Diagnosis Scale – preliminary revision. Autism in Adulthood, 2(1), 34–41. 10.1089/aut.2019.0059 36600983 PMC8992847

[bibr9-13623613251347740] Ashwood K. L. Gillan N. Horder J. Hayward H. Woodhouse E. McEwen F. S. Findon J. Eklund H. Spain D. Wilson C. E. Cadman T. Young S. Stoencheva V. Murphy C. M. Robertson D. Charman T. Bolton P. Glaser K. Asherson P. Murphy D. G. (2016). Predicting the diagnosis of autism in adults using the Autism-Spectrum Quotient (AQ) questionnaire. Psychological Medicine, 46(12), 2595–2604. 10.1017/s0033291716001082 27353452 PMC4988267

[bibr10-13623613251347740] Austin E. J. (2005). Personality correlates of the broader autism phenotype as assessed by the Autism Spectrum Quotient (AQ). Personality and Individual Differences, 38(2), 451–460. 10.1016/j.paid.2004.04.022

[bibr11-13623613251347740] Aykan S. Gürses E. Tokgöz-Yılmaz S. Kalaycıoğlu C. (2020). Auditory processing differences correlate with autistic traits in males. Frontiers in Human Neuroscience, 14, Article 584704. 10.3389/fnhum.2020.584704 PMC7588834 33192419

[bibr12-13623613251347740] Bailey A. Palferman S. Heavey L. Le Couteur A. (1998). Autism: The phenotype in relatives. Journal of Autism and Developmental Disorders, 28(5), 369–392. 10.1023/A:1026048320785 9813774

[bibr13-13623613251347740] Baron-Cohen S. Ashwin E. Ashwin C. Tavassoli T. Chakrabarti B. (2011). The paradox of autism: Why does disability sometimes give rise to talent? 10.1017/CBO9780511978098.017

[bibr14-13623613251347740] Baron-Cohen S. Wheelwright S. Skinner R. Martin J. Clubley E. (2001). The Autism-Spectrum Quotient (AQ): Evidence from Asperger syndrome/high-functioning autism, males and females, scientists and mathematicians. Journal of Autism and Developmental Disorders, 31(1), 5–17. 10.1023/a:1005653411471 11439754

[bibr15-13623613251347740] Belcher H. L. Morein-Zamir S. Stagg S. D. Ford R. M. (2022). Shining a light on a hidden population: Social functioning and mental health in women reporting autistic traits but lacking diagnosis. Journal of Autism and Developmental Disorders, 53(8), 3118–3132. 10.1007/s10803-022-05583-2 35593995 PMC10313531

[bibr16-13623613251347740] Bernaards C. A. Jennrich R. I. (2005). Gradient projection algorithms and software for arbitrary rotation criteria in factor analysis. Educational and Psychological Measurement, 65(5), 676–696. 10.1177/0013164404272507

[bibr17-13623613251347740] Bervoets J. Hens K. (2020). Going beyond the catch-22 of autism diagnosis and research. The moral implications of (not) asking ‘what is autism?’ Frontiers in Psychology, 11, Article 529193. 10.3389/fpsyg.2020.529193 PMC7683513 33240143

[bibr18-13623613251347740] Bezemer M. L. Blijd-Hoogewys E. M. A. Meek-Heekelaar M. (2021). The predictive value of the AQ and the SRS-A in the diagnosis of ASD in adults in clinical practice. Journal of Autism and Developmental Disorders, 51(7), 2402–2415. 10.1007/s10803-020-04699-7 33001348 PMC8189953

[bibr19-13623613251347740] Bijlenga D. Tjon-Ka-Jie J. Y. M. Schuijers F. Kooij J. J. S. (2017). Atypical sensory profiles as core features of adult ADHD, irrespective of autistic symptoms. European Psychiatry, 43, 51–57. 10.1016/j.eurpsy.2017.02.481 28371743

[bibr20-13623613251347740] Bölte S. Neufeld J. Marschik P. B. Williams Z. J. Gallagher L. Lai M.-C. (2023). Sex and gender in neurodevelopmental conditions. Nature Reviews Neurology, 1–24. 10.1038/s41582-023-00774-6 PMC10154737 36747038

[bibr21-13623613251347740] Botha M. Dibb B. Frost D. M. (2020). ‘Autism is me’: An investigation of how autistic individuals make sense of autism and stigma. Disability & Society, 37, 427–453. 10.1080/09687599.2020.1822782

[bibr22-13623613251347740] Bottema-Beutel K. Kapp S. K. Lester J. N. Sasson N. J. Hand B. N. (2021). Avoiding ableist language: Suggestions for autism researchers. Autism in Adulthood, 3(1), 18–29. 10.1089/aut.2020.0014 36601265 PMC8992888

[bibr23-13623613251347740] Bralten J. van Hulzen K. J. Martens M. B. Galesloot T. E. Arias Vasquez A. Kiemeney L. A. Buitelaar J. K. Muntjewerff J. W. Franke B. Poelmans G. (2018). Autism spectrum disorders and autistic traits share genetics and biology. Molecular Psychiatry, 23(5), Article 5. 10.1038/mp.2017.98 PMC5984081 28507316

[bibr24-13623613251347740] Brett J. D. Peden B. Preece D. A. Whitehouse A. Becerra R. Maybery M. T. (2024). Assessing restricted and repetitive behaviours in online-sampled autistic and non-autistic individuals: Factor structure of the Repetitive Behaviours Questionnaire for Adults (RBQ-2A). Journal of Autism and Developmental Disorders, 54(6), 2138–2147. 10.1007/s10803-023-05977-w 37052863 PMC11142953

[bibr25-13623613251347740] Cerda N. Brinster M. Turner C. Shahidullah J. D. Augustyn M. (2023). Challenging case: Leveraging community partnerships to address barriers to care for students with autism. Journal of Developmental & Behavioral Pediatrics, 44(3), e239–e241. 10.1097/dbp.0000000000001163 36716769

[bibr26-13623613251347740] Chahboun S. Stenseng F. Page A. G. (2022). The changing faces of autism: The fluctuating international diagnostic criteria and the resulting inclusion and exclusion – A Norwegian perspective. Frontiers in Psychiatry, 13, Article 787893. 10.3389/fpsyt.2022.787893 PMC9365987 35966490

[bibr27-13623613251347740] Chen F. F. (2007). Sensitivity of goodness of fit indexes to lack of measurement invariance. Structural Equation Modeling: A Multidisciplinary Journal, 14(3), 464–504. 10.1080/10705510701301834

[bibr28-13623613251347740] Cheung G. W. Rensvold R. B. (2002). Evaluating goodness-of-fit indexes for testing measurement invariance. Structural Equation Modeling: A Multidisciplinary Journal, 9(2), 233–255. 10.1207/S15328007SEM0902_5

[bibr29-13623613251347740] Cohen J. (2013). Statistical power analysis for the behavioral sciences. Academic Press.

[bibr30-13623613251347740] Conner C. M. Cramer R. D. McGonigle J. J. (2019). Examining the diagnostic validity of autism measures among adults in an outpatient clinic sample. Autism in Adulthood, 1(1), 60–68. 10.1089/aut.2018.0023 36600688 PMC8992806

[bibr31-13623613251347740] Constantino J. N. (2012). Social Responsiveness Scale Second Edition (SRS-2): Manual. Western Psychological Services (WPS).

[bibr32-13623613251347740] Constantino J. N. Davis S. A. Todd R. D. Schindler M. K. Gross M. M. Brophy S. L. Metzger L. M. Shoushtari C. S. Splinter R. Reich W. (2003). Validation of a brief quantitative measure of autistic traits: Comparison of the Social Responsiveness Scale with the autism diagnostic interview-revised. Journal of Autism and Developmental Disorders, 33(4), 427–433. 10.1023/A:1025014929212 12959421

[bibr33-13623613251347740] Crawshaw D. (2023). Should we continue to tell autistic people that their brains are different? Psychological Reports, 128, 1315–1355. 10.1177/00332941231174391 37147123 PMC11977825

[bibr34-13623613251347740] Dababnah S. Shaia W. E. Campion K. Nichols H. M. (2018). ‘We had to keep pushing’: Caregivers’ perspectives on autism screening and referral practices of Black children in primary care. Intellectual and Developmental Disabilities, 56(5), 321–336. 10.1352/1934-9556-56.5.321 30273522

[bibr35-13623613251347740] Davies J. Cooper K. Killick E. Sam E. Healy M. Thompson G. Mandy W. Redmayne B. Crane L. (2023). Autistic identity: A systematic review of quantitative research. Autism Research, 17, 874–897. 10.1002/aur.3105 38334318

[bibr36-13623613251347740] Davis A. Solomon M. Belcher H. (2022). Examination of race and autism intersectionality among African American/Black young adults. Autism in Adulthood, 4(4), 306–314. 10.1089/aut.2021.0091 36777378 PMC9908282

[bibr37-13623613251347740] D’Mello A. M. Frosch I. R. Li C. E. Cardinaux A. L. Gabrieli J. D. E. (2022). Exclusion of females in autism research: Empirical evidence for a ‘leaky’ recruitment-to-research pipeline. Autism Research, 15(10), 1929–1940. 10.1002/aur.2795 36054081 PMC9804357

[bibr38-13623613251347740] Dorlack T. P. Myers O. B. Kodituwakku P. W. (2018). A comparative analysis of the ADOS-G and ADOS-2 algorithms: Preliminary findings. Journal of Autism and Developmental Disorders, 48(6), 2078–2089. 10.1007/s10803-018-3475-3 29380271

[bibr39-13623613251347740] English M. C. W. Gignac G. E. Visser T. A. W. Whitehouse A. J. O. Enns J. T. Maybery M. T. (2021). The Comprehensive Autistic Trait Inventory (CATI): Development and validation of a new measure of autistic traits in the general population. Molecular Autism, 12(1), 37. 10.1186/s13229-021-00445-7 PMC8130295 34001225

[bibr40-13623613251347740] English M. C. W. Gignac G. E. Visser T. A. W. Whitehouse A. J. O. Maybery M. T. (2020). A comprehensive psychometric analysis of autism-spectrum quotient factor models using two large samples: Model recommendations and the influence of divergent traits on total-scale scores. Autism Research, 13(1), 45–60. 10.1002/aur.2198 31464106

[bibr41-13623613251347740] Fletcher-Watson S. (2023). What’s in a name? The costs and benefits of a formal autism diagnosis. Autism, 28, 257–262. 10.1177/13623613231213300 37997793

[bibr42-13623613251347740] Fletcher-Watson S. Adams J. Brook K. Charman T. Crane L. Cusack J. Leekam S. Milton D. Parr J. R. Pellicano E. (2019). Making the future together: Shaping autism research through meaningful participation. Autism, 23(4), 943–953. 10.1177/1362361318786721 30095277 PMC6512245

[bibr43-13623613251347740] Glidden D. Bouman W. P. Jones B. A. Arcelus J. (2016). Gender Dysphoria and autism spectrum disorder: A systematic review of the literature. Sexual Medicine Reviews, 4(1), 3–14. 10.1016/j.sxmr.2015.10.003 27872002

[bibr44-13623613251347740] Goodman M. Adams N. Corneil T. Kreukels B. Motmans J. Coleman E. (2019). Size and distribution of transgender and gender nonconforming populations: A narrative review. Endocrinology and Metabolism Clinics of North America, 48(2), 303–321. 10.1016/j.ecl.2019.01.001 31027541

[bibr45-13623613251347740] Gunderson J. Worthley E. Byiers B. Symons F. Wolff J. (2023). Self and caregiver report measurement of sensory features in autism spectrum disorder: A systematic review of psychometric properties. Journal of Neurodevelopmental Disorders, 15, 5. 10.1186/s11689-022-09473-7 36698071 PMC9875408

[bibr46-13623613251347740] Hechler F. C. Tuomainen O. Weber N. Fahr F. Karlek B. Maroske M. Misia M. Caruana N. (2025). ‘What does “often” even mean?’ Revising and validating the Comprehensive Autistic Trait Inventory in partnership with autistic people. Molecular Autism, 16(1), 7. 10.1186/s13229-025-00643-7 39915887 PMC11803966

[bibr47-13623613251347740] Hilton M. N. Boulton K. A. Kozlowska K. McClure G. Guastella A. J. (2022). The co-occurrence of neurodevelopmental disorders in gender dysphoria: Characteristics within a paediatric treatment-seeking cohort and factors that predict distress pertaining to gender. Journal of Psychiatric Research, 149, 281–286. 10.1016/j.jpsychires.2022.02.018 35306277

[bibr48-13623613251347740] Hu L. Bentler P. M. (1999). Cutoff criteria for fit indexes in covariance structure analysis: Conventional criteria versus new alternatives. Structural Equation Modeling: A Multidisciplinary Journal, 6(1), 1–55. 10.1080/10705519909540118

[bibr49-13623613251347740] Huang Y. Arnold S. R. C. Foley K.-R. Trollor J. N. (2022a). Experiences of support following autism diagnosis in adulthood. Journal of Autism and Developmental Disorders, 54, 518–531. 10.1007/s10803-022-05811-9 36409392

[bibr50-13623613251347740] Huang Y. Arnold S. R. C. Foley K.-R. Trollor J. N. (2022b). A qualitative study of adults’ and support persons’ experiences of support after autism diagnosis. Journal of Autism and Developmental Disorders, 54, 1157–1170. 10.1007/s10803-022-05828-0 36484961 PMC9734854

[bibr51-13623613251347740] Hume R. Burgess H. (2021). ‘I’m human after all’: Autism, trauma, and affective empathy. Autism in Adulthood, 3(3), 221–229. 10.1089/aut.2020.0013 36605372 PMC8992898

[bibr52-13623613251347740] Hurley R. S. E. Losh M. Parlier M. Reznick J. S. Piven J. (2007). The Broad Autism Phenotype Questionnaire. Journal of Autism and Developmental Disorders, 37(9), 1679–1690. 10.1007/s10803-006-0299-3 17146701

[bibr53-13623613251347740] The jamovi project. (2024). Jamovi (Version 2.5) [Computer software]. https://www.jamovi.org/

[bibr54-13623613251347740] Jarrold C. Brock J. (2004). To match or not to match? Methodological issues in autism-related research. Journal of Autism and Developmental Disorders, 34(1), 81–86. 10.1023/B:JADD.0000018078.82542.ab 15098961

[bibr55-13623613251347740] Johnston K. Dittner A. Bramham J. Murphy C. Knight A. Russell A. (2013). ADHD symptoms in adult ASD. Autism Research, 6(4), 225–236. 10.1002/aur.1283 23788522

[bibr56-13623613251347740] Jones L. Goddard L. Hill E. L. Henry L. A. Crane L. (2014). Experiences of receiving a diagnosis of autism spectrum disorder: A survey of adults in the United Kingdom. Journal of Autism and Developmental Disorders, 44(12), 3033–3044. 10.1007/s10803-014-2161-3 24915932

[bibr57-13623613251347740] Jones S. C. (2022). Measuring the wrong thing the right way? Time to rethink autism research tools. Autism in Adulthood, 4(2), 104–109. 10.1089/aut.2021.0050 36605973 PMC9645667

[bibr58-13623613251347740] Jones S. L. Johnson M. Alty B. Adamou M. (2021). The effectiveness of RAADS-R as a screening tool for adult ASD populations. Autism Research and Treatment, 2021, 9974791. 10.1155/2021/9974791 PMC8452438 34552768

[bibr59-13623613251347740] Jorgensen T. D. Pornprasertmanit S. Schoemann A. M. Rosseel Y. Miller P. Quick C. Garnier-Villarreal M. Selig J. Boulton A. Preacher K. Coffman D. Rhemtulla M. Robitzsch A. Enders C. Arslan R. Clinton B. Panko P. Merkle E. Chesnut S. . . .Johnson A. R. (2022). semTools: Useful tools for structural equation modeling (Version 0.5-6) [Computer software]. https://cran.r-project.org/web/packages/semTools/index.html

[bibr60-13623613251347740] Kallitsounaki A. Williams D. M. (2023). Autism spectrum disorder and gender dysphoria/incongruence. A systematic literature review and meta-analysis. Journal of Autism and Developmental Disorders, 53(8), 3103–3117. 10.1007/s10803-022-05517-y 35596023 PMC10313553

[bibr61-13623613251347740] Kanner L. (1944). Early infantile autism. The Journal of Pediatrics, 25(3), 211–217. 10.1016/S0022-3476(44)80156-1

[bibr62-13623613251347740] Kapp S. Gillespie-Lynch K. Sherman L. Hutman T. (2013). Deficit, difference, or both? Autism and neurodiversity. Developmental Psychology, 49, 59–71. 10.1037/a0028353 22545843

[bibr63-13623613251347740] Kuiper M. W. Verhoeven E. W. Geurts H. M. (2019). The Dutch Glasgow Sensory Questionnaire: Psychometric properties of an autism-specific sensory sensitivity measure. Autism, 23(4), 922–932. 10.1177/1362361318788065 30073851

[bibr64-13623613251347740] Lai M.-C. Lombardo M. V. Auyeung B. Chakrabarti B. Baron-Cohen S. (2015). Sex/gender differences and autism: Setting the scene for future research. Journal of the American Academy of Child & Adolescent Psychiatry, 54(1), 11–24. 10.1016/j.jaac.2014.10.003 25524786 PMC4284309

[bibr65-13623613251347740] Landry O. Chouinard P. A. (2016). Why we should study the broader autism phenotype in typically developing populations. Journal of Cognition and Development, 17(4), 584–595. 10.1080/15248372.2016.1200046

[bibr66-13623613251347740] Leedham A. Thompson A. R. Smith R. Freeth M. (2019). ‘I was exhausted trying to figure it out’: The experiences of females receiving an autism diagnosis in middle to late adulthood. Autism, 24(1), 135–146. 10.1177/1362361319853442 31144507

[bibr67-13623613251347740] Levy S. E. Mandell D. S. Schultz R. T. (2009). Autism. The Lancet, 374(9701), 1627–1638. 10.1016/S0140-6736(09)61376-3 PMC2863325 19819542

[bibr68-13623613251347740] Lewis L. F. (2016). Exploring the experience of self-diagnosis of autism spectrum disorder in adults. Archives of Psychiatric Nursing, 30(5), 575–580. 10.1016/j.apnu.2016.03.009 27654240

[bibr69-13623613251347740] Lewis L. F. (2017). A mixed methods study of barriers to formal diagnosis of autism spectrum disorder in adults. Journal of Autism and Developmental Disorders, 47(8), 2410–2424. 10.1007/s10803-017-3168-3 28516422

[bibr70-13623613251347740] Lilley R. Lawson W. Hall G. Mahony J. Clapham H. Heyworth M. Arnold S. R. Trollor J. N. Yudell M. Pellicano E. (2021). ‘A way to be me’: Autobiographical reflections of autistic adults diagnosed in mid-to-late adulthood. Autism, 26(6), 1395–1408. 10.1177/13623613211050694 34674564

[bibr71-13623613251347740] Lockwood Estrin G. Milner V. Spain D. Happé F. Colvert E . (2021). Barriers to autism spectrum disorder diagnosis for young women and girls: A systematic review. Review Journal of Autism and Developmental Disorders, 8(4), 454–470. 10.1007/s40489-020-00225-8 34868805 PMC8604819

[bibr72-13623613251347740] Lundin Remnélius K. Bölte S . (2023). Camouflaging in autism: Age effects and cross-cultural validation of the Camouflaging Autistic Traits Questionnaire (CAT-Q). Journal of Autism and Developmental Disorders, 1–16. 10.1007/s10803-023-05909-8 PMC11136743 36757540

[bibr73-13623613251347740] Lundström S. Chang Z. Råstam M. Gillberg C. Larsson H. Anckarsäter H. Lichtenstein P. (2012). Autism spectrum disorders and autisticlike traits: Similar etiology in the extreme end and the normal variation. Archives of General Psychiatry, 69(1), 46–52. 10.1001/archgenpsychiatry.2011.144 22213788

[bibr74-13623613251347740] Lyall K. (2023). What are quantitative traits and how can they be used in autism research? Autism Research, 16(7), 1289–1298. 10.1002/aur.2937 37212172 PMC10524676

[bibr75-13623613251347740] Malik-Soni N. Shaker A. Luck H. Mullin A. E. Wiley R. E. Lewis M. E. S. Fuentes J. Frazier T. W. (2022). Tackling healthcare access barriers for individuals with autism from diagnosis to adulthood. Pediatric Research, 91(5), Article 5. 10.1038/s41390-021-01465-y PMC7993081 33767375

[bibr76-13623613251347740] Markon K. E. (2019). Bifactor and hierarchical models: Specification, inference, and interpretation. Annual Review of Clinical Psychology, 15, 51–69. 10.1146/annurev-clinpsy-050718-095522 30649927

[bibr77-13623613251347740] McDonald T. A. M. (2020). Autism identity and the ‘lost generation’: Structural validation of the autism spectrum identity scale and comparison of diagnosed and self-diagnosed adults on the autism spectrum. Autism in Adulthood, 2(1), 13–23. 10.1089/aut.2019.0069 34485832 PMC8415774

[bibr78-13623613251347740] McFarland D. J. (2016). Modeling general and specific abilities: Evaluation of bifactor models for the WJ-III. Assessment, 23(6), 698–706. 10.1177/1073191115595070 26187901

[bibr79-13623613251347740] McKenzie K. Forsyth K. O’Hare A. McClure I. Rutherford M. Murray A. Irvine L. (2015). Factors influencing waiting times for diagnosis of Autism Spectrum Disorder in children and adults. Research in Developmental Disabilities, 45–46, 300–306. 10.1016/j.ridd.2015.07.033 26280693

[bibr80-13623613251347740] Miller D. Rees J. Pearson A. (2021). ‘Masking is life’: Experiences of masking in autistic and nonautistic adults. Autism in Adulthood: Challenges and Management, 3(4), 330–338. 10.1089/aut.2020.0083 36601640 PMC8992921

[bibr81-13623613251347740] Mosner M. G. Kinard J. L. Shah J. S. McWeeny S. Greene R. K. Lowery S. C. Mazefsky C. A. Dichter G. S. (2019). Rates of co-occurring psychiatric disorders in autism spectrum disorder using the mini international neuropsychiatric interview. Journal of Autism and Developmental Disorders, 49(9), 3819–3832. 10.1007/s10803-019-04090-1 31175504 PMC6669096

[bibr82-13623613251347740] Murray D. Lesser M. Lawson W. (2005). Attention, monotropism and the diagnostic criteria for autism. Autism, 9(2), 139–156. 10.1177/1362361305051398 15857859

[bibr83-13623613251347740] Nicolaidis C. Raymaker D. McDonald K. Dern S. Ashkenazy E. Boisclair C. Robertson S. Baggs A. (2011). Collaboration strategies in nontraditional community-based participatory research partnerships: Lessons from an academic–community partnership with autistic self-advocates. Progress in Community Health Partnerships: Research, Education, and Action, 5, 143–150. 10.1353/cpr.2011.0022 21623016 PMC3319698

[bibr84-13623613251347740] Nishiyama T. Suzuki M. Adachi K. Sumi S. Okada K. Kishino H. Sakai S. Kamio Y. Kojima M. Suzuki S. Kanne S. M. (2014). Comprehensive Comparison of Self-administered Questionnaires for measuring quantitative autistic traits in adults. Journal of Autism and Developmental Disorders, 44(5), 993–1007. 10.1007/s10803-013-2020-7 24342972

[bibr85-13623613251347740] Nobili A. Glazebrook C. Bouman W. P. Glidden D. Baron-Cohen S. Allison C. Smith P. Arcelus J. (2018). Autistic traits in treatment-seeking transgender adults. Journal of Autism and Developmental Disorders, 48(12), 3984–3994. 10.1007/s10803-018-3557-2 29654452 PMC6223809

[bibr86-13623613251347740] Pearson A. Rose K. (2021). A conceptual analysis of autistic masking: Understanding the narrative of stigma and the illusion of choice. Autism in Adulthood, 3(1), 52–60. 10.1089/aut.2020.0043 36601266 PMC8992880

[bibr87-13623613251347740] Pellicano E. Lawson W. Hall G. Mahony J. Lilley R. Heyworth M. Clapham H. Yudell M. (2021). ‘I knew she’d get it, and get me’: Participants’ Perspectives of a participatory autism research project. Autism in Adulthood, 4. 10.1089/aut.2021.0039 PMC9645671 36605972

[bibr88-13623613251347740] Posit Team. (2023). RStudio: Integrated development environment for R. Posit Software, PBC. https://www.posit.co/

[bibr89-13623613251347740] Poulsen R. Brownlow C. Lawson W. Pellicano E. (2022). Meaningful research for autistic people? Ask autistics! Autism, 26(1), 3–5. 10.1177/13623613211064421 35000419

[bibr90-13623613251347740] Poulsen R. Tan D. W. Sowman P. F. McAlpine D. Pellicano E. (2025). Auditory environments influence the link between Autistic traits and quality of life. Scientific Reports, 15(1), 10612. 10.1038/s41598-025-94585-y 40148426 PMC11950209

[bibr91-13623613251347740] R Core Team. (2022). R: A language and environment for statistical computing. R Foundation for Statistical Computing. https://www.r-project.org/

[bibr92-13623613251347740] Revelle W. (2024). psych: Procedures for psychological, psychometric, and personality research (Version 2.4.12) [Computer software]. https://cran.r-project.org/web/packages/psych/index.html

[bibr93-13623613251347740] Ritvo R. A. Ritvo E. R. Guthrie D. Ritvo M. J. Hufnagel D. H. McMahon W. Tonge B. Mataix-Cols D. Jassi A. Attwood T. Eloff J. (2011). The Ritvo Autism Asperger Diagnostic Scale-Revised (RAADS-R): A scale to assist the diagnosis of autism spectrum disorder in adults: An international validation study. Journal of Autism and Developmental Disorders, 41(8), 1076–1089. 10.1007/s10803-010-1133-5 21086033 PMC3134766

[bibr94-13623613251347740] Robertson A. E. Simmons D. R. (2013). The relationship between sensory sensitivity and autistic traits in the general population. Journal of Autism and Developmental Disorders, 43(4), 775–784. 10.1007/s10803-012-1608-7 22832890

[bibr95-13623613251347740] Robin X. Turck N. Hainard A. Tiberti N. Lisacek F. Sanchez J.-C. Müller M. (2011). pROC: An open-source package for R and S+ to analyze and compare ROC curves. BMC Bioinformatics, 12(1), 77. 10.1186/1471-2105-12-77 21414208 PMC3068975

[bibr96-13623613251347740] Robinson E. B. St Pourcain B. Anttila V. Kosmicki J. A. Bulik-Sullivan B. Grove J. Maller J. Samocha K. E. Sanders S. J. Ripke S. Martin J. Hollegaard M. V. Werge T. Hougaard D. M. iPSYCH-SSI-Broad Autism Group, Neale B. M. Evans D. M. Skuse D. Mortensen P. B. Børglum A. D. . . .Daly M. J. (2016). Genetic risk for autism spectrum disorders and neuropsychiatric variation in the general population. Nature Genetics, 48(5), 552–555. 10.1038/ng.3529 PMC4986048 26998691

[bibr97-13623613251347740] Rosseel Y. (2012). Lavaan: An R package for structural equation modeling. Journal of Statistical Software, 48, 1–36. 10.18637/jss.v048.i02

[bibr98-13623613251347740] Rutter M. (2000). Genetic studies of autism: From the 1970s into the millennium. Journal of Abnormal Child Psychology, 28(1), 3–14. 10.1023/A:1005113900068 10772346

[bibr99-13623613251347740] Ruzich E. Allison C. Smith P. Watson P. Auyeung B. Ring H. Baron-Cohen S. (2015). Subgrouping siblings of people with autism: Identifying the broader autism phenotype. Autism Research, 9(6), 658–665. 10.1002/aur.1544 26332889 PMC4915503

[bibr100-13623613251347740] Sasson N. J. Bottema-Beutel K. (2022). Studies of autistic traits in the general population are not studies of autism. Autism, 26(4), 1007–1008. 10.1177/13623613211058515 34825582

[bibr101-13623613251347740] Silberman S. (2015). NeuroTribes: The legacy of autism and the future of neurodiversity. Avery Publishing. https://www.amazon.com/NeuroTribes-Legacy-Autism-Future-Neurodiversity/dp/0399185615

[bibr102-13623613251347740] Smith-Young J. Chafe R. Audas R. (2020). ‘Managing the wait’: Parents’ experiences in accessing diagnostic and treatment services for children and adolescents diagnosed with autism spectrum disorder. Health Services Insights, 13, 1178632920902141. 10.1177/1178632920902141 PMC6987484 32063709

[bibr103-13623613251347740] Streiner D. L. (2003). Starting at the beginning: An introduction to coefficient alpha and internal consistency. Journal of Personality Assessment, 80(1), 99–103. 10.1207/S15327752JPA8001_18 12584072

[bibr104-13623613251347740] Sturm A. Huang S. Bal V. Schwartzman B. (2024). Psychometric exploration of the RAADS-R with autistic adults: Implications for research and clinical practice. Autism, 28(9), 2334–2345. 10.1177/13623613241228329 38305196

[bibr105-13623613251347740] Sucksmith E. Roth I. Hoekstra R. A. (2011). Autistic traits below the clinical threshold: Re-examining the broader autism phenotype in the 21st century. Neuropsychology Review, 21(4), 360–389. 10.1007/s11065-011-9183-9 21989834

[bibr106-13623613251347740] Taber K. S. (2018). The use of Cronbach’s alpha when developing and reporting research instruments in science education. Research in Science Education, 48(6), 1273–1296. 10.1007/s11165-016-9602-2

[bibr107-13623613251347740] Tavakol M. Dennick R. (2011). Making sense of Cronbach’s alpha. International Journal of Medical Education, 2, 53–55. 10.5116/ijme.4dfb.8dfd 28029643 PMC4205511

[bibr108-13623613251347740] Taylor E. Holt R. Tavassoli T. Ashwin C. Baron-Cohen S. (2020). Revised scored Sensory Perception Quotient reveals sensory hypersensitivity in women with autism. Molecular Autism, 11(1), 18. 10.1186/s13229-019-0289-x 32122389 PMC7053068

[bibr109-13623613251347740] Warrier V. Greenberg D. M. Weir E. Buckingham C. Smith P. Lai M.-C. Allison C. Baron-Cohen S. (2020). Elevated rates of autism, other neurodevelopmental and psychiatric diagnoses, and autistic traits in transgender and gender-diverse individuals. Nature Communications, 11(1), 3959. 10.1038/s41467-020-17794-1 PMC7415151 32770077

[bibr110-13623613251347740] Wigham S. Rodgers J. Berney T. Le Couteur A. Ingham B. Parr J. R. (2019). Psychometric properties of questionnaires and diagnostic measures for autism spectrum disorders in adults: A systematic review. Autism, 23(2), 287–305. 10.1177/1362361317748245 29439585

[bibr111-13623613251347740] Wu A. D. Li Z. Zumbo B. D. (2007). Decoding the meaning of factorial invariance and updating the practice of multi-group confirmatory factor analysis: A demonstration with TIMSS data. Practical Assessment, Research, and Evaluation, 12(1), Article 1. 10.7275/mhqa-cd89

[bibr112-13623613251347740] Yu Y. Ozonoff S. Miller M. (2023). Assessment of autism spectrum disorder. Assessment, 31, 24–41. 10.1177/10731911231173089 37248660 PMC10676043

[bibr113-13623613251347740] Zeidan J. Fombonne E. Scorah J. Ibrahim A. Durkin M. S. Saxena S. Yusuf A. Shih A. Elsabbagh M. (2022). Global prevalence of autism: A systematic review update. Autism Research, 15(5), 778–790. 10.1002/aur.2696 35238171 PMC9310578

