# Supplementary material for: Psychometric evaluation of the Comprehensive Autistic Trait Inventory in autistic and non-autistic adults
Source: Autism. 2025 Jul 16;29(12):2955–74. doi: 10.1177/13623613251347740 (PMC12618716; doi:10.1177/13623613251347740)
Supplement: sj-docx-1-aut-10.1177_13623613251347740 – Supplemental material for Psychometric evaluation of the Comprehensive Autistic Trait Inventory in autistic and non-autistic adults [file sj-docx-1-aut-10.1177_13623613251347740.docx]

**Psychometric evaluation of the Comprehensive Autistic Trait Inventory (CATI) in autistic and non-autistic adults**

Michael CW English*, Rebecca E Poulsen*, Murray T Maybery, David McAlpine,
Paul F Sowman, Elizabeth Pellicano

* Co-first authors

**SUPPLEMENTARY MATERIAL**

[**Table S1.** Demographic information for participants, including age, diagnostic status, and gender identity, separated by dataset. Numbers in parentheses refer to standard deviation around mean age. 2](#_Toc201563415)

[**Table S2.** Results of multiple-group factorial analyses assessing measurement invariance of the Sensory Sensitivity subscale of the CATI comparing participants who were administered the scale with the original (Dataset 1 & 2)) and revised wording (Dataset 3 & 4) of three sensory sensitivity items. 3](#_Toc201563416)

[**Table S3.** Responses to ‘Gender’ for gender diverse participants (n = 336). 3](#_Toc201563417)

[**Table S4.** Responses to questions relating to participant ethnicity. As participants may select more than choice, the percentages do not sum to 100%. 4](#_Toc201563418)

[**Table S5.** Pearson correlations with 95% confidence intervals (10,000 bootstrapped samples) between CATI subscales for total sample and separated by participant group. 5](#_Toc201563419)

[**Table S6.** Additional descriptive statistics describing CATI total-scale and subscale scores for the overall sample and separated by autism group. 7](#_Toc201563420)

[**Table S7.** Model summaries, including standardised coefficients, of a logistic regression analysis predicting autism status (i.e., Autistic DX vs non-Autistic) using the CATI higher-order subscale scores for the total sample and separately for gender identity subgroups. 8](#_Toc201563421)

[**Table S8.** Model summaries, including standardised coefficients, of a logistic regression analysis predicting autism status (i.e., Autistic DX vs non-Autistic) using the CATI six subscale scores for the total sample and separately for gender identity subgroups. 9](#_Toc201563422)

[**Table S9.** Pearson correlations with 95% confidence intervals (10,000 bootstrapped samples) between CATI subscales for total sample and separated by gender. 10](#_Toc201563423)

[**Table S10.** Additional descriptive statistics describing CATI total-scale and subscale scores for the overall sample and separated by autism group and gender. 11](#_Toc201563424)

**Table S1.** Demographic information for participants, including age, diagnostic status, and gender identity, separated by dataset. Numbers in parentheses refer to standard deviation around mean age.

|  | **All participants** | **Non-autistic** | **Autistic _ALL_** | **Autistic _DX_** | **Autistic _SELF_** |
| --- | --- | --- | --- | --- | --- |
| ***Dataset 1: Study 2 within English et al. (2021)*** | | | | | |
| **N** | 1113 | 999 | 114 | 45 | 69 |
| **Gender** |  |  |  |  |  |
| Cisgender man | 551 | 495 | 56 | 20 | 36 |
| Cisgender woman | 541 | 495 | 46 | 18 | 28 |
| Gender diverse ^a^ | 21 | 9 | 12 | 7 | 5 |
| Gender not given | 0 | 0 | 0 | 0 | 0 |
| **Mean Age [*SD*]** | 37.0 [12.6] | 37.5 [12.6] | 32.4 [11.6] | 28.5 [9.4] | 34.9 [12.2] |
| ***Dataset 2: Study 2 within Brett et al. (2023)*** | | | | | |
| **N** | 268 | 4 | 264 | 261 | 3 |
| **Gender** |  |  |  |  |  |
| Cisgender man | 122 | 3 | 119 | 118 | 1 |
| Cisgender woman | 115 | 1 | 114 | 112 | 2 |
| Gender diverse ^a^ | 31 | 0 | 31 | 31 | 0 |
| Gender not given | 0 | 0 | 0 | 0 | 0 |
| **Mean Age [*SD*]** | 34.8 [14.6] | 34.8 [14.6] | 32.4 [10.2] | 32.4 [10.3] | 34.3 [5.0] |
| ***Dataset 3: Poulsen et al. (2025)*** | | | | | |
| **N** | 381 | 0 | 0 | 263 | 118 |
| **Gender** |  |  |  |  |  |
| Cisgender man | 51 | 0 | 51 | 32 | 19 |
| Cisgender woman | 238 | 0 | 238 | 162 | 76 |
| Gender diverse ^a^ | 91 | 0 | 91 | 68 | 23 |
| Gender not given | 1 | 0 | 1 | 1 | 0 |
| **Mean Age [*SD*]** | 38.3 [10.9] | - | 38.3 [10.9] | 38.0 [10.6] | 38.9 [11.4] |
| ***Dataset 4: Additional data collected for current study*** | | | | | |
| **N** | 839 | 276 | 563 | 168 | 395 |
| **Gender** |  |  |  |  |  |
| Cisgender man | 481 | 147 | 334 | 142 | 192 |
| Cisgender woman | 165 | 60 | 105 | 5 | 100 |
| Gender diverse ^a^ | 193 | 69 | 124 | 21 | 103 |
| Gender not given | 0 | 0 | 0 | 0 | 0 |
| **Mean Age [*SD*]** | 35.3 [11.3] | 37.7 [34.1] | 34.1 [10.9] | 32.0 [9.8] | 34.9 [11.2] |

^a^ Gender diverse is inclusive of identities such as, but not limited to, nonbinary, gender queer, demigender, agender, and transgender. See Table S2 for further detail.

**Table S2.** Results of multiple-group factorial analyses assessing measurement invariance of the Sensory Sensitivity subscale of the CATI comparing participants who were administered the scale with the original (Dataset 1 & 2)) and revised wording (Dataset 3 & 4) of three sensory sensitivity items.

| **Model** | **χ^2^** | ***df*** | **CFI** | **RMSEA (90% CI)** | **Δχ^2^** | **Δ*df*** | **ΔCFI** | **ΔRMSEA** | **Decision** |
| --- | --- | --- | --- | --- | --- | --- | --- | --- | --- |
| **Configural Invariance** | 371 | 28 | .980 | .062 (.054-.072) | - | - | - | - | Accept |
| **Metric (weak) invariance** | 419 | 48 | .979 | .054 (.047-.061) | 48 | 20 | .001 | .008 | Accept |
| **Scalar (strong) invariance** | 446 | 54 | .978 | .060 (.053-.066) | 27 | 6 | .001 | -.006 | Accept |
| **Residual (strict) invariance** | 421 | 61 | .979 | .057 (.050-.063) | -25 | 7 | -.001 | .003 | Accept |

Note: ΔCFI > 0.01 and ΔRMSEA > 0.015 indicate a violation of the invariance assumption. CFI: comparative fit index; RMSEA: root mean square error of approximation (Chen, 2007; Cheung & Rensvold, 2002).

**Table S3.** Responses to ‘Gender’ for gender diverse participants (n = 336).

| **Response** | **Count (%)** | **Response** | **Count (%)** |
| --- | --- | --- | --- |
| Nonbinary | 213 (63.4%) | Transgender | 1 (0.3%) |
| Agender | 19 (5.7%) | Nonbinary/genderqueer | 1 (0.3%) |
| Trans - Female / Woman | 17 (5.5%) | Nonbinary, transmasculine, bigender | 1 (0.3%) |
| Trans - Male / Man | 11 (3.3%) | Agender/transmasculine | 1 (0.3%) |
| Genderqueer | 12 (3.6%) | None/agender/transfeminine | 1 (0.3%) |
| Genderfluid | 10 (3.0%) | Transgender man / Transmasculine nonbinary | 1 (0.3%) |
| Nonbinary man | 4 (1.2%) | Undetermined | 1 (0.3%) |
| Nonbinary woman | 4 (1.2%) | Genderqueer woman | 1 (0.3%) |
| Nonbinary/agender | 3 (0.9%) | Trans masc non binary | 1 (0.3%) |
| Nonbinary transmasculine | 3 (0.9%) | Bigender | 1 (0.3%) |
| Nonbinary Trans Man | 2 (0.6%) | Genderfluid/nonbinary | 1 (0.3%) |
| I do not have a gender identity | 2 (0.6%) | Genderqueer transmasc | 1 (0.3%) |
| Demi-gender woman | 2 (0.6%) | Gender Void - lacking a sense of, or connection to, gender | 1 (0.3%) |
| gender apathetic | 1 (0.3%) | No sense of gender - present transmasculine | 1 (0.3%) |
| Genderfluid woman | 1 (0.3%) | Queer | 1 (0.3%) |
| Gender diverse / genderfluid / genderqueer | 1 (0.3%) | Trans masculine | 1 (0.3%) |
| Woman but don't mind she or they pronouns | 1 (0.3%) | Nonbinary - transgender | 1 (0.3%) |
| Nonbinary transmasculine person | 1 (0.3%) | Nonbinary/questioning | 1 (0.3%) |
| Female/ genderqueer | 1 (0.3%) | Variable | 1 (0.3%) |
| Male/Nonbinary (Fluid) | 1 (0.3%) | *Gender Diverse (no additional detail)* | 8 (2.4%) |

**Table S4.** Responses to questions relating to participant ethnicity. As participants may select more than choice, the percentages do not sum to 100%.

| **Ethnicity** | **Count** | **Percentage** |
| --- | --- | --- |
| **White (Caucasian/European)** | **2103** | **80.85%** |
| White (Caucasian) | 2050 | 78.82% |
| European (Eastern) | 17 | 0.65% |
| European (Mainland) | 36 | 1.38% |
| **Indigenous Populations** | **140** | **5.39%** |
| Indigenous Australian or Torres Strait Islander | 112 | 4.31% |
| Māori or Indigenous New Zealander | 8 | 0.31% |
| American Indian / Native American or Alaskan Native | 16 | 0.62% |
| Native Hawaiian or Pacific Islander | 4 | 0.15% |
| **Asian** | 123 | 4.73% |
| Asian (unspecified) | 74 | 2.85% |
| South Asian / East Asian | 42 | 1.61% |
| Indian (unspecified) | 7 | 0.27% |
| **Hispanic, Latino or Spanish** | **67** | **2.58%** |
| **Black / African Descent** | 79 | 3.04% |
| Black/African American | 59 | 2.27% |
| African (unspecified) | 20 | 0.77% |
| **Middle Eastern / North African** | 14 | 0.54% |
| **Caribbean** | **15** | **0.58%** |
| **Mixed** | **36** | **1.38%** |
| **Other / Unspecified** | **174** | **6.69%** |

Note: As the present study combined samples from several independently run studies that varied in how ethnicity was recorded, meaning the level of specificity in the data presented in this table also varies.

**Table S5.** Pearson correlations with 95% confidence intervals (10,000 bootstrapped samples) between CATI subscales for total sample and separated by participant group.

|  | **Social (higher order)** | **Non-social (higher order)** | **Social Interaction** | **Communication** | **Social Camouflage** | **Self-Regulatory Behaviour** | **Cognitive Flexibility** |
| --- | --- | --- | --- | --- | --- | --- | --- |
| **All participants (N = 2,601)** | | | | | | | |
| Non-social (higher order) | .72 [.70 - .74] |  |  |  |  |  |  |
| Social Interaction | .86 [.85 - .87] | .54 [.51 - .57] |  |  |  |  |  |
| Communication | .81 [.80 - .82] | .58 [.56 - .61] | .55 [.52 - .58] |  |  |  |  |
| Social Camouflage | .83 [.82 - .84] | .68 [.66 - .70] | .57 [.54 - .60] | .52 [.49 - .55] |  |  |  |
| Self-Regulatory Behaviour | .62 [.59 - .64] | .88 [.87 - .89] | .44 [.41 - .47] | .50 [.47 - .53] | .62 [.59 - .64] |  |  |
| Cognitive Flexibility | .59 [.56 - .62] | .83 [.81 - .84] | .44 [.41 - .48] | .48 [.45 - .51] | .56 [.53 - .59] | .60 [.58 - .63] |  |
| Sensory Sensitivity | .64 [.62 - .67] | .88 [.87 - .89] | .51 [.48 - .54] | .52 [.49 - .54] | .59 [.56 - .61] | .64 [.62 - .67] | .59 [.57 - .62] |
| **Non-autistic (n = 1,279)** | | | | | | | |
| Non-social (higher order) | .57 [.53 - .61] |  |  |  |  |  |  |
| Social Interaction | .86 [.85 - .87] | .39 [.34 - .44] |  |  |  |  |  |
| Communication | .71 [.68 - .73] | .43 [.38 - .48] | .41 [.36 - .46] |  |  |  |  |
| Social Camouflage | .79 [.77 - .81] | .56 [.52 - .60] | .50 [.46 - .54] | .41 [.36 - .45] |  |  |  |
| Self-Regulatory Behaviour | .47 [.43 - .52] | .83 [.81 - .84] | .30 [.25 - .35] | .36 [.31 - .41] | .49 [.45 - .54] |  |  |
| Cognitive Flexibility | .41 [.36 - .46] | .77 [.74 - .79] | .26 [.20 - .31] | .33 [.28 - .38] | .42 [.37 - .46] | .47 [.43 - .52] |  |
| Sensory Sensitivity | .48 [.44 - .53] | .81 [.79 - .83] | .38 [.33 - .43] | .34 [.29 - .39] | .44 [.39 - .48] | .48 [.43 - .53] | .44 [.39 - .49] |

*Note: All correlations p < .001, except when marked with* * *(p < .01). …continues over page…*

|  | **Social (higher order)** | **Non-social (higher order)** | **Social Interaction** | **Communication** | **Social Camouflage** | **Self-Regulatory Behaviour** | **Cognitive Flexibility** |
| --- | --- | --- | --- | --- | --- | --- | --- |
| **Autistic _ALL_ (n = 1,322)** | | | | | | | |
| Non-social (higher order) | .52 [.47-.56] |  |  |  |  |  |  |
| Social Interaction | .79 [.76-.81] | .34 [.28-.39] |  |  |  |  |  |
| Communication | .74 [.72-.77] | .33 [.28-.38] | .41 [.36-.46] |  |  |  |  |
| Social Camouflage | .70 [.67-.73] | .49 [.44-.54] | .34 [.28-.39] | .23 [.17-.28] |  |  |  |
| Self-Regulatory Behaviour | .39 [.34-.44] | .82 [.80-.84] | .20 [.14-.26] | .24 [.19-.30] | .43 [.38-.49] |  |  |
| Cognitive Flexibility | .47 [.42-.52] | .78 [.75-.81] | .34 [.29-.40] | .30 [.25-.35] | .41 [.36-.46] | .49 [.44-.54] |  |
| Sensory Sensitivity | .41 [.36-.46] | .83 [.81-.85] | .29 [.22-.35] | .26 [.21-.32] | .37 [.31-.42] | .51 [.46-.56] | .48 [.42-.53] |
| **Autistic _DX_ (n = 737)** | | | | | | | |
| Non-social (higher order) | .58 [.53-.64] |  |  |  |  |  |  |
| Social Interaction | .82 [.79-.85] | .42 [.35-.49] |  |  |  |  |  |
| Communication | .75 [.72-.78] | .37 [.30-.44] | .48 [.42-.54] |  |  |  |  |
| Social Camouflage | .69 [.65-.73] | .53 [.47-.60] | .37 [.30-.44] | .22 [.14-.29] |  |  |  |
| Self-Regulatory Behaviour | .45 [.37-.51] | .83 [.81-.86] | .27 [.18-.35] | .28 [.21-.35] | .47 [.39-.54] |  |  |
| Cognitive Flexibility | .54 [.48-.60] | .80 [.76-.83] | .43 [.36-.50] | .34 [.27-.41] | .46 [.39-.53] | .53 [.45-.59] |  |
| Sensory Sensitivity | .47 [.40-.54] | .84 [.82-.87] | .35 [.27-.43] | .31 [.23-.38] | .40 [.33-.47] | .54 [.47-.60] | .51 [.43-.58] |
| **Autistic _SELF_ (n = 585)** | | | | | | | |
| Non-social (higher order) | .42 [.34-.49] |  |  |  |  |  |  |
| Social Interaction | .73 [.69-.77] | .22 [.13-.30] |  |  |  |  |  |
| Communication | .74 [.70-.78] | .26 [.18-.34] | .33 [.25-.40] |  |  |  |  |
| Social Camouflage | .70 [.66-.74] | .44 [.37-.51] | .29 [.20-.37] | .25 [.17-.33] |  |  |  |
| Self-Regulatory Behaviour | .32 [.23-.40] | .81 [.78-.84] | .12 [.03-.21] * | .18 [.09-.27] | .39 [.30-.47] |  |  |
| Cognitive Flexibility | .37 [.29-.45] | .76 [.71-.79] | .21 [.13-.30] | .24 [.17-.32] | .34 [.26-.42] | .44 [.36-.51] |  |
| Sensory Sensitivity | .32 [.25-.40] | .82 [.78-.84] | .19 [.11-.27] | .20 [.12-.28] | .32 [.24-.39] | .47 [.40-.54] | .43 [.35-.51] |

*Note: All correlations p < .001, except when marked with* * *(p < .01).*

**Table S6.** Additional descriptive statistics describing CATI total-scale and subscale scores for the overall sample and separated by autism group.

|  | **Mean** | **Stand. Dev.** | **25^th^ perc.** | **50^th^ perc.** | **75^th^ perc.** |
| --- | --- | --- | --- | --- | --- |
| **All participants (N = 2,601)** |  |  |  |  |  |
| Total-scale | 138.77 | 32.92 | 116.00 | 142.00 | 165.00 |
| Social (higher order) | 66.48 | 17.70 | 54.00 | 68.00 | 80.00 |
| Non-social (higher order) | 72.29 | 17.84 | 60.00 | 74.00 | 87.00 |
| Social Interactions | 25.09 | 7.81 | 20.00 | 27.00 | 32.00 |
| Communication | 18.51 | 6.49 | 13.00 | 18.00 | 23.00 |
| Social Camouflage | 22.87 | 6.85 | 18.00 | 23.00 | 28.00 |
| Self-Regulatory Behaviour | 23.21 | 7.23 | 18.00 | 24.00 | 29.00 |
| Cognitive Flexibility | 25.90 | 5.85 | 22.00 | 27.00 | 30.00 |
| Sensory Sensitivity | 23.18 | 7.59 | 17.00 | 24.00 | 29.00 |
| **Non-Autistic (n = 1,279)** |  |  |  |  |  |
| Total-scale | 116.73 | 26.89 | 97.00 | 119.00 | 136.00 |
| Social (higher order) | 55.37 | 15.15 | 44.00 | 56.00 | 66.00 |
| Non-social (higher order) | 61.36 | 15.20 | 51.00 | 62.00 | 72.00 |
| Social Interactions | 21.40 | 7.99 | 15.00 | 22.00 | 28.00 |
| Communication | 14.88 | 5.01 | 11.00 | 14.00 | 18.00 |
| Social Camouflage | 19.10 | 5.97 | 15.00 | 19.00 | 23.50 |
| Self-Regulatory Behaviour | 19.36 | 6.74 | 14.00 | 19.00 | 24.00 |
| Cognitive Flexibility | 23.16 | 5.52 | 19.00 | 23.00 | 27.00 |
| Sensory Sensitivity | 18.84 | 6.66 | 14.00 | 19.00 | 24.00 |
| **Autistic _ALL_ (n = 1,322)** |  |  |  |  |  |
| Total-scale | 160.09 | 22.55 | 148.00 | 163.00 | 176.00 |
| Social (higher order) | 77.22 | 12.63 | 70.00 | 79.00 | 86.00 |
| Non-social (higher order) | 82.87 | 13.23 | 76.00 | 85.00 | 92.00 |
| Social Interactions | 28.66 | 5.70 | 25.00 | 30.00 | 33.00 |
| Communication | 22.03 | 5.78 | 18.00 | 22.00 | 26.00 |
| Social Camouflage | 26.52 | 5.55 | 23.00 | 27.00 | 31.00 |
| Self-Regulatory Behaviour | 26.94 | 5.53 | 24.00 | 28.00 | 31.00 |
| Cognitive Flexibility | 28.55 | 4.84 | 26.00 | 29.00 | 32.00 |
| Sensory Sensitivity | 27.38 | 5.88 | 24.00 | 29.00 | 32.00 |
| **Autistic _DX_ (n = 737)** |  |  |  |  |  |
| Total-scale | 160.58 | 24.07 | 150.00 | 164.00 | 177.00 |
| Social (higher order) | 77.20 | 13.27 | 70.00 | 79.00 | 87.00 |
| Non-social (higher order) | 83.38 | 13.77 | 76.00 | 86.00 | 93.00 |
| Social Interactions | 28.24 | 5.99 | 25.00 | 30.00 | 33.00 |
| Communication | 22.53 | 5.83 | 19.00 | 23.00 | 27.00 |
| Social Camouflage | 26.43 | 5.71 | 23.00 | 27.00 | 31.00 |
| Self-Regulatory Behaviour | 27.24 | 5.56 | 24.00 | 28.00 | 31.00 |
| Cognitive Flexibility | 28.57 | 5.01 | 26.00 | 29.00 | 32.00 |
| Sensory Sensitivity | 27.56 | 6.08 | 24.00 | 29.00 | 32.00 |
| **Autistic _SELF_ (n = 585)** |  |  |  |  |  |
| Total-scale | 159.47 | 20.48 | 147.00 | 162.00 | 175.00 |
| Social (higher order) | 77.24 | 11.79 | 70.00 | 78.00 | 86.00 |
| Non-social (higher order) | 82.23 | 12.52 | 76.00 | 84.00 | 91.00 |
| Social Interactions | 29.20 | 5.25 | 26.00 | 30.00 | 34.00 |
| Communication | 21.40 | 5.67 | 17.00 | 21.00 | 25.00 |
| Social Camouflage | 26.64 | 5.36 | 23.00 | 28.00 | 31.00 |
| Self-Regulatory Behaviour | 26.56 | 5.46 | 24.00 | 27.00 | 30.00 |
| Cognitive Flexibility | 28.53 | 4.63 | 26.00 | 29.00 | 32.00 |
| Sensory Sensitivity | 27.14 | 5.62 | 24.00 | 28.00 | 31.00 |

**Table S7.** Model summaries, including standardised coefficients, of a logistic regression analysis predicting autism status (i.e., Autistic DX vs non-Autistic) using the CATI higher-order subscale scores for the total sample and separately for gender identity subgroups.

|  |  | **Wald Test** | |  | **95% CI for Odds Ratio** | |
| --- | --- | --- | --- | --- | --- | --- |
|  | **β** | **Wald** | ***p*** | **Odds Ratio** | **Lower** | **Upper** |
| **Total Sample** | | | | | | |
| ***df* = 2, χ^2^ = 1018, *p* < .001, Nagelkerke’s pseudo *R^2^* = .542, AUC = .888** | | | | | | |
| (Intercept) | -9.788 | 522 | < .001 | 0.000 | 0.000 | 0.000 |
| *Social higher-order subscale* | 0.066 | 147 | < .001 | 1.069 | 1.058 | 1.081 |
| *Non-social higher-order subscale* | 0.065 | 146 | < .001 | 1.067 | 1.056 | 1.078 |
| **Cisgender man** | | | | | | |
| ***df* = 2, χ^2^ = 315, *p* < .001, Nagelkerke’s pseudo *R^2^* = .391, AUC = .830** | | | | | | |
| (Intercept) | -7.729 | 217 | < .001 | 0.000 | 0.000 | 0.001 |
| *Social higher-order subscale* | 0.059 | 60 | < .001 | 1.060 | 1.045 | 1.076 |
| *Non-social higher-order subscale* | 0.044 | 38 | < .001 | 1.045 | 1.031 | 1.060 |
| **Cisgender woman** | | | | | | |
| ***df* = 2, χ^2^ = 556, *p* < .001, Nagelkerke’s pseudo *R^2^* = .660, AUC = .929** | | | | | | |
| (Intercept) | -12.180 | 223 | < .001 | 0.000 | 0.000 | 0.000 |
| *Social higher-order subscale* | 0.080 | 72 | < .001 | 1.084 | 1.064 | 1.104 |
| *Non-social higher-order subscale* | 0.083 | 76 | < .001 | 1.087 | 1.067 | 1.107 |
| **Gender Diverse** | | | | | | |
| ***df* = 2, χ^2^ = 116, *p* < .001, Nagelkerke’s pseudo *R^2^* = .587, AUC = .909** | | | | | | |
| (Intercept) | -13.871 | 44 | < .001 | 0.000 | 0.000 | 0.000 |
| *Social higher-order subscale* | 0.067 | 14 | < .001 | 1.070 | 1.033 | 1.176 |
| *Non-social higher-order subscale* | 0.117 | 25 | < .001 | 1.124 | 1.074 | 1.176 |

**Table S8.** Model summaries, including standardised coefficients, of a logistic regression analysis predicting autism status (i.e., Autistic DX vs non-Autistic) using the CATI six subscale scores for the total sample and separately for gender identity subgroups.

|  |  | **Wald Test** | |  | **95% CI for Odds Ratio** | |
| --- | --- | --- | --- | --- | --- | --- |
|  | **β** | **Wald** | ***p*** | **Odds Ratio** | **Lower** | **Upper** |
| **Total Sample** | | | | | | |
| ***df* = 6, χ^2^ = 1098, *p* < .001, Nagelkerke’s pseudo *R^2^* = .574, AUC = .899** | | | | | | |
| (Intercept) | -9.306 | 439 | < .001 | 0.000 | 0.000 | 0.000 |
| *Social Interaction* | 0.000 | 0 | .947 | 1.001 | 0.980 | 1.022 |
| *Communication* | 0.150 | 133 | < .001 | 1.161 | 1.132 | 1.191 |
| *Social Camouflage* | 0.083 | 37 | < .001 | 1.087 | 1.058 | 1.116 |
| *Self-Regulatory Behaviour* | 0.063 | 24 | < .001 | 1.065 | 1.038 | 1.093 |
| *Cognitive Flexibility* | 0.010 | 0 | .522 | 1.010 | 0.980 | 1.041 |
| *Sensory Sensitivity* | 0.096 | 62 | < .001 | 1.101 | 1.075 | 1.128 |
| **Cisgender man** | | | | | | |
| ***df* = 6, χ^2^ = 342, *p* < .001, Nagelkerke’s pseudo *R^2^* = .419, AUC = .840** | | | | | | |
| (Intercept) | -7.490 | 184 | < .001 | 0.000 | 0.000 | 0.000 |
| *Social Interaction* | 0.011 | 1 | .443 | 1.011 | 0.984 | 1.038 |
| *Communication* | 0.123 | 49 | < .001 | 1.131 | 1.093 | 1.171 |
| *Social Camouflage* | 0.075 | 17 | < .001 | 1.078 | 1.040 | 1.118 |
| *Self-Regulatory Behaviour* | 0.033 | 4 | .059 | 1.034 | 0.999 | 1.070 |
| *Cognitive Flexibility* | 0.007 | 0 | .728 | 1.007 | 0.967 | 1.049 |
| *Sensory Sensitivity* | 0.070 | 17 | < .001 | 1.073 | 1.037 | 1.109 |
| **Cisgender woman** | | | | | | |
| ***df* = 6, χ^2^ = 604, *p* < .001, Nagelkerke’s pseudo *R^2^* = .700, AUC = .941** | | | | | | |
| (Intercept) | -11.404 | 183 | < .001 | 0.000 | 0.000 | 0.000 |
| *Social Interaction* | -0.016 | 1 | .447 | 0.984 | 0.944 | 1.026 |
| *Communication* | 0.187 | 58 | < .001 | 1.206 | 1.149 | 1.265 |
| *Social Camouflage* | 0.106 | 19 | < .001 | 1.112 | 1.060 | 1.166 |
| *Self-Regulatory Behaviour* | 0.114 | 24 | < .001 | 1.121 | 1.071 | 1.172 |
| *Cognitive Flexibility* | -0.022 | 1 | .423 | 0.979 | 0.929 | 1.032 |
| *Sensory Sensitivity* | 0.133 | 33 | < .001 | 1.142 | 1.091 | 1.195 |
| **Gender Diverse** | | | | | | |
| ***df* = 6, χ^2^ = 131, *p* < .001, Nagelkerke’s pseudo *R^2^* = .642, AUC = .922** | | | | | | |
| (Intercept) | -12.477 | 33 | < .001 | 0.000 | 0.000 | 0.000 |
| *Social Interaction* | -0.029 | 0 | .496 | 0.972 | 0.895 | 1.055 |
| *Communication* | 0.208 | 20 | < .001 | 1.232 | 1.124 | 1.350 |
| *Social Camouflage* | 0.028 | 0 | .566 | 1.028 | 0.935 | 1.130 |
| *Self-Regulatory Behaviour* | 0.048 | 1 | .345 | 1.049 | 0.950 | 1.159 |
| *Cognitive Flexibility* | 0.139 | 7 | .007 | 1.150 | 1.038 | 1.273 |
| *Sensory Sensitivity* | 0.142 | 8 | .004 | 1.153 | 1.048 | 1.268 |

**Table S9.** Pearson correlations with 95% confidence intervals (10,000 bootstrapped samples) between CATI subscales for total sample and separated by gender.

|  | **Social (higher order)** | **Non-social (higher order)** | **Social Interaction** | **Communication** | **Social Camouflage** | **Self-Regulatory Behaviour** | **Cognitive Flexibility** |
| --- | --- | --- | --- | --- | --- | --- | --- |
| **Cisgender Man (n = 1,205)** | | | | | | | |
| Non-social (higher order) | .69 [.65-.72] |  |  |  |  |  |  |
| Social Interaction | .86 [.85-.88] | .51 [.46-.55] |  |  |  |  |  |
| Communication | .79 [.76-.81] | .54 [.50-.58] | .53 [.48-.57] |  |  |  |  |
| Social Camouflage | .78 [.76-.80] | .65 [.61-.68] | .50 [.46-.55] | .43 [.38-.48] |  |  |  |
| Self-Regulatory Behaviour | .57 [.53-.61] | .86 [.84-.87] | .39 [.34-.44] | .43 [.38-.48] | .59 [.55-.63] |  |  |
| Cognitive Flexibility | .55 [.50-.59] | .81 [.78-.83] | .42 [.37-.47] | .42 [.38-.47] | .50 [.46-.55] | .55 [.51-.59] |  |
| Sensory Sensitivity | .62 [.58-.66] | .87 [.85-.88] | .48 [.43-.52] | .52 [.48-.57] | .54 [.49-.58] | .60 [.56-.64] | .56 [.52-.60] |
| **Cisgender Woman (n = 1,059)** | | | | | | | |
| Non-social (higher order) | .74 [.71-.77] |  |  |  |  |  |  |
| Social Interaction | .88 [.86-.89] | .59 [.54-.63] |  |  |  |  |  |
| Communication | .83 [.82-.85] | .63 [.59-.66] | .59 [.55-.62] |  |  |  |  |
| Social Camouflage | .87 [.86-.89] | .70 [.66-.73] | .65 [.61-.69] | .61 [.57-.64] |  |  |  |
| Self-Regulatory Behaviour | .65 [.61-.68] | .88 [.87-.90] | .49 [.44-.54] | .56 [.52-.60] | .63 [.59-.67] |  |  |
| Cognitive Flexibility | .61 [.57-.65] | .84 [.82-.86] | .47 [.42-.52] | .53 [.48-.57] | .58 [.54-.63] | .64 [.60-.67] |  |
| Sensory Sensitivity | .67 [.63-.70] | .89 [.87-.90] | .56 [.52-.61] | .55 [.51-.59] | .62 [.58-.65] | .66 [.62-.70] | .63 [.58-.66] |
| **Gender Diverse (n = 336)** | | | | | | | |
| Non-social (higher order) | .61 [.51-.69] |  |  |  |  |  |  |
| Social Interaction | .78 [.72-.83] | .31 [.16-.44] |  |  |  |  |  |
| Communication | .81 [.77-.84] | .53 [.45-.60] | .46 [.36-.55] |  |  |  |  |
| Social Camouflage | .78 [.72-.82] | .60 [.49-.68] | .41 [.29-.52] | .44 [.33-.53] |  |  |  |
| Self-Regulatory Behaviour | .52 [.41-.61] | .87 [.82-.90] | .25 [.11-.38] | .45 [.37-.53] | .52 [.42-.61] |  |  |
| Cognitive Flexibility | .57 [.46-.66] | .84 [.79-.88] | .32 [.18-.44] | .47 [.38-.56] | .55 [.44-.65] | .59 [.48-.68] |  |
| Sensory Sensitivity | .47 [.35-.57] | .85 [.81-.89] | .23 [.08-.36] | .43 [.33-.52] | .45 [.33-.56] | .62 [.52-.70] | .56 [.45-.66] |

*Note: All correlations p < .001.*

**Table S10.** Additional descriptive statistics describing CATI total-scale and subscale scores for the overall sample and separated by autism group and gender.

|  | **Non-autistic** | | | | | **Autistic** | | | | |
| --- | --- | --- | --- | --- | --- | --- | --- | --- | --- | --- |
|  | **Mean** | **Std. Dev.** | **25^th^ perc.** | **50^th^ perc.** | **75^th^ perc.** | **Mean** | **Std. Dev.** | **25^th^ perc.** | **50^th^ perc.** | **75^th^ perc.** |
| **Cisgender Man (n = 1,205)** |  |  |  |  |  |  |  |  |  |  |
| Total-scale | 118.29 | 26.26 | 99.00 | 120.00 | 138.00 | 153.08 | 24.11 | 139.00 | 156.00 | 169.00 |
| Social (higher order) | 56.66 | 14.73 | 46.00 | 57.00 | 67.00 | 74.78 | 13.17 | 67.00 | 76.00 | 84.00 |
| Non-social (higher order) | 61.64 | 14.80 | 51.00 | 62.00 | 72.00 | 78.30 | 14.08 | 70.00 | 80.00 | 89.00 |
| Social Interactions | 21.34 | 7.94 | 15.00 | 22.00 | 28.00 | 27.98 | 6.27 | 24.00 | 29.50 | 33.00 |
| Communication | 15.96 | 5.13 | 12.00 | 15.00 | 19.00 | 21.77 | 5.82 | 18.00 | 22.00 | 26.00 |
| Social Camouflage | 19.35 | 5.72 | 15.00 | 19.00 | 24.00 | 25.03 | 5.69 | 22.00 | 26.00 | 29.00 |
| Self-Reg. Behaviour | 20.06 | 6.74 | 15.00 | 21.00 | 25.00 | 25.72 | 5.87 | 22.00 | 27.00 | 30.00 |
| Cognitive Flexibility | 23.55 | 5.28 | 20.00 | 24.00 | 27.00 | 27.79 | 5.20 | 25.00 | 28.00 | 32.00 |
| Sensory Sensitivity | 18.03 | 6.28 | 13.00 | 18.00 | 23.00 | 24.79 | 6.21 | 21.00 | 26.00 | 29.00 |
| **Cisgender Woman (n = 1,059)** |  |  |  |  |  |  |  |  |  |  |
| Total-scale | 113.11 | 26.64 | 93.00 | 114.00 | 132.00 | 163.54 | 20.89 | 152.00 | 166.00 | 178.00 |
| Social (higher order) | 53.16 | 15.24 | 41.75 | 52.50 | 64.00 | 78.65 | 12.10 | 72.00 | 80.00 | 87.00 |
| Non-social (higher order) | 59.95 | 15.08 | 49.00 | 60.00 | 70.00 | 84.89 | 12.23 | 79.00 | 87.00 | 93.00 |
| Social Interactions | 20.96 | 8.03 | 14.00 | 21.00 | 27.00 | 29.15 | 5.10 | 26.00 | 30.00 | 33.00 |
| Communication | 13.65 | 4.47 | 10.00 | 13.00 | 16.00 | 21.99 | 5.82 | 18.00 | 22.00 | 26.00 |
| Social Camouflage | 18.55 | 6.09 | 14.00 | 19.00 | 23.00 | 27.50 | 5.18 | 25.00 | 28.00 | 31.00 |
| Self-Reg. Behaviour | 18.07 | 6.42 | 13.00 | 18.00 | 23.00 | 27.13 | 5.33 | 24.00 | 28.00 | 31.00 |
| Cognitive Flexibility | 22.74 | 5.63 | 19.00 | 23.00 | 27.00 | 28.75 | 4.70 | 27.00 | 29.00 | 32.00 |
| Sensory Sensitivity | 19.13 | 6.82 | 14.00 | 19.00 | 23.00 | 29.01 | 5.09 | 26.00 | 30.00 | 33.00 |
| **Gender Diverse (n = 336)** |  |  |  |  |  |  |  |  |  |  |
| Total-scale | 129.58 | 28.97 | 113.00 | 136.00 | 148.00 | 168.46 | 17.12 | 157.00 | 170.50 | 180.75 |
| Social (higher order) | 60.50 | 15.66 | 52.00 | 62.00 | 71.00 | 79.68 | 11.54 | 73.00 | 81.00 | 88.00 |
| Non-social (higher order) | 69.08 | 17.04 | 64.25 | 75.00 | 79.00 | 88.78 | 9.38 | 84.00 | 90.00 | 96.00 |
| Social Interactions | 24.92 | 7.29 | 21.00 | 25.00 | 31.00 | 29.17 | 5.35 | 26.00 | 30.00 | 33.00 |
| Communication | 14.64 | 5.63 | 10.00 | 14.50 | 19.00 | 22.69 | 5.60 | 19.00 | 23.00 | 27.00 |
| Social Camouflage | 20.94 | 6.70 | 16.00 | 22.00 | 26.75 | 27.81 | 5.20 | 25.00 | 29.00 | 32.00 |
| Self-Reg. Behaviour | 22.77 | 7.03 | 18.00 | 24.50 | 28.00 | 29.20 | 4.24 | 27.00 | 30.00 | 32.00 |
| Cognitive Flexibility | 22.83 | 6.50 | 19.00 | 24.00 | 27.75 | 29.79 | 3.94 | 28.00 | 30.00 | 33.00 |
| Sensory Sensitivity | 23.47 | 6.52 | 19.00 | 25.00 | 28.75 | 29.79 | 4.21 | 28.00 | 30.00 | 33.00 |
