# Supplementary material for: Psychometric evaluation of the Comprehensive Autistic Trait Inventory in autistic and non-autistic adults
Source: Autism. 2025 Jul 16;29(12):2955–74. doi: 10.1177/13623613251347740 (PMC12618716; doi:10.1177/13623613251347740)
Supplement: sj-zip-1-aut-10.1177_13623613251347740 – Supplemental material for Psychometric evaluation of the Comprehensive Autistic Trait Inventory in autistic and non-autistic adults [file sj-zip-1-aut-10.1177_13623613251347740.zip › CATI - basic.pdf]

CATI

Date: Name/ID:

INSTRUCTIONS

Below is a list of statements relating to various personality traits, behaviours, and characteristics. Using the five response options select the option that best describes you. For items of a social nature, think about situations that do not involve very close friends or family members. Try not to spend too much time thinking about each choice.

		Definitely Disagree	Somewhat Disagree	Neither Agree nor Disagree	Somewhat Agree	Definitely Agree
**1**	**I often find myself fiddling or playing repetitively with objects (e.g. clicking pens)**					
**2**	**I like to stick to certain routines for every-day tasks**					
**3**	**I expend a lot of mental energy trying to fit in with others**					
**4**	**I am very sensitive to bright lighting**					
**5**	**There are certain activities that I always choose to do the same way, every time**					
**6**	**Sometimes I watch people interacting and try to copy them when I need to socialise**					
**7**	**I often rock when sitting in a chair**					
**8**	**I generally enjoy social events**					
**9**	**I look for strategies and ways to appear more sociable**					
**10**	**In social situations, I try to avoid interactions with other people**					
**11**	**There are times when I feel that my senses are overloaded**					
**12**	**There are certain objects that I fiddle or play with that can help me calm down or collect my thoughts**					
**13**	**Reading non-verbal cues (e.g. facial expressions, body language) is difficult for me**					
**14**	**I like my belongings to be sorted in certain ways and will spend time making sure they are that way**					
**15**	**Social interaction is easy for me**					
**16**	**When interacting with other people, I spend a lot of effort monitoring how I am coming across**					
**17**	**I find social interactions stressful**					
**18**	**I am very sensitive to touch**					
**19**	**I can tell how people feel from their facial expressions**					
		**Definitely Disagree**	**Somewhat Disagree**	**Neither Agree nor Disagree**	**Somewhat Agree**	**Definitely Agree**

		Definitely Disagree	Somewhat Disagree	Neither Agree nor Disagree	Somewhat Agree	Definitely Agree
20	I have a tendency to pace or move around in a repetitive path					
21	I feel discomfort when prevented from completing a particular routine					
22	I rely on a set of scripts when I talk with people					
23	I find it easy to sense what someone else is feeling					
24	I am very sensitive to particular tastes (e.g. salty, sour, spicy, or sweet)					
25	I engage in certain repetitive actions when I feel stressed					
26	I rarely use non-verbal cues in my interactions with others					
27	I often insist on doing things in a certain way, or re-doing things until they are 'just right'					
28	I feel confident or capable when meeting new people					
29	Before engaging in a social situation, I will create a script to follow where possible					
30	Social occasions are often challenging for me					
31	Sometimes the presence of a smell makes it hard for me to focus on anything else					
32	There are certain repetitive actions that others consider to be 'characteristic' of me (e.g. stroking my hair)					
33	Metaphors or 'figures of speech' often confuse me					
34	It annoys me when plans I have made are changed					
35	I find it difficult to make new friends					
36	I react strongly to unexpected loud noises					
37	I have difficulty understanding someone else's point-of-view					
38	I like to arrange items in rows or patterns					
39	I try to follow certain 'rules' in order to get by in social situations					
40	I am sensitive to flickering lights					
41	I have certain habits that I find difficult to stop (e.g. biting/tearing nails, pulling strands of hair)					
42	I have difficulty understanding the 'unspoken rules' of social situations					
		Definitely Disagree	Somewhat Disagree	Neither Agree nor Disagree	Somewhat Agree	Definitely Agree

ADMIN ONLY						
TOTAL	SOC	COM	CAM	FLX	REG	SEN
