# Supplementary material for: Psychometric evaluation of the Comprehensive Autistic Trait Inventory in autistic and non-autistic adults
Source: Autism. 2025 Jul 16;29(12):2955–74. doi: 10.1177/13623613251347740 (PMC12618716; doi:10.1177/13623613251347740)
Supplement: sj-zip-1-aut-10.1177_13623613251347740 – Supplemental material for Psychometric evaluation of the Comprehensive Autistic Trait Inventory in autistic and non-autistic adults [file sj-zip-1-aut-10.1177_13623613251347740.zip › CATI - scoring key.pdf]

CATI

SCORING KEY

All items are scored 1 to 5 ('Definitely Disagree' to 'Definitely Agree') except items 8, 15, 19, 23, and 28 (highlighted below), which are reversed. Total scale score ranges from 42 – 210, whilst subscales range 7 – 35.

The six subscales are each made up of seven items. The subscale for each item is listed in the second column.

SOC = Social Interactions
COM = Communication
CAM = Social Camouflage
RIG = Cognitive (In)Flexibility
REG = Self-regulatory Behaviours
SEN = Sensory Sensitivity

			Definitely Disagree	Somewhat Disagree	Neither Agree nor Disagree	Somewhat Agree	Definitely Agree
1	REG	I often find myself fiddling or playing repetitively with objects (e.g. clicking pens)	1	2	3	4	5
2	FLX	I like to stick to certain routines for every-day tasks	1	2	3	4	5
3	CAM	I expend a lot of mental energy trying to fit in with others	1	2	3	4	5
4	SEN	I am very sensitive to bright lighting	1	2	3	4	5
5	FLX	There are certain activities that I always choose to do the same way, every time	1	2	3	4	5
6	CAM	Sometimes I watch people interacting and try to copy them when I need to socialise	1	2	3	4	5
7	REG	I often rock when sitting in a chair	1	2	3	4	5
8	SOC	I generally enjoy social events	5	4	3	2	1
9	CAM	I look for strategies and ways to appear more sociable	1	2	3	4	5
10	SOC	In social situations, I try to avoid interactions with other people	1	2	3	4	5
11	SEN	There are times when I feel that my senses are overloaded	1	2	3	4	5
12	REG	There are certain objects that I fiddle or play with that can help me calm down or collect my thoughts	1	2	3	4	5
13	COM	Reading non-verbal cues (e.g. facial expressions, body language) is difficult for me	1	2	3	4	5
14	FLX	I like my belongings to be sorted in certain ways and will spend time making sure they are that way	1	2	3	4	5
15	SOC	Social interaction is easy for me	5	4	3	2	1
16	CAM	When interacting with other people, I spend a lot of effort monitoring how I am coming across	1	2	3	4	5
17	SOC	I find social interactions stressful	1	2	3	4	5
18	SEN	I am very sensitive to touch	1	2	3	4	5
19	COM	I can tell how people feel from their facial expressions	5	4	3	2	1
			Definitely Disagree	Somewhat Disagree	Neither Agree nor Disagree	Somewhat Agree	Definitely Agree

			Definitely Disagree	Somewhat Disagree	Neither Agree nor Disagree	Somewhat Agree	Definitely Agree
20	REG	I have a tendency to pace or move around in a repetitive path	1	2	3	4	5
21	FLX	I feel discomfort when prevented from completing a particular routine	1	2	3	4	5
22	CAM	I rely on a set of scripts when I talk with people	1	2	3	4	5
23	COM	I find it easy to sense what someone else is feeling	5	4	3	2	1
24	SEN	I am very sensitive to particular tastes (e.g. salty, sour, spicy, or sweet)	1	2	3	4	5
25	REG	I engage in certain repetitive actions when I feel stressed	1	2	3	4	5
26	COM	I rarely use non-verbal cues in my interactions with others	1	2	3	4	5
27	FLX	I often insist on doing things in a certain way, or re-doing things until they are 'just right'	1	2	3	4	5
28	SOC	I feel confident or capable when meeting new people	5	4	3	2	1
29	CAM	Before engaging in a social situation, I will create a script to follow where possible	1	2	3	4	5
30	SOC	Social occasions are often challenging for me	1	2	3	4	5
31	SEN	Sometimes the presence of a smell makes it hard for me to focus on anything else	1	2	3	4	5
32	REG	There are certain repetitive actions that others consider to be 'characteristic' of me (e.g. stroking my hair)	1	2	3	4	5
33	COM	Metaphors or 'figures of speech' often confuse me	1	2	3	4	5
34	FLX	It annoys me when plans I have made are changed	1	2	3	4	5
35	SOC	I find it difficult to make new friends	1	2	3	4	5
36	SEN	I react strongly to unexpected loud noises	1	2	3	4	5
37	COM	I have difficulty understanding someone else's point-of-view	1	2	3	4	5
38	FLX	I like to arrange items in rows or patterns	1	2	3	4	5
39	CAM	I try to follow certain 'rules' in order to get by in social situations	1	2	3	4	5
40	SEN	I am sensitive to flickering lights	1	2	3	4	5
41	REG	I have certain habits that I find difficult to stop (e.g. biting/tearing nails, pulling strands of hair)	1	2	3	4	5
42	COM	I have difficulty understanding the 'unspoken rules' of social situations	1	2	3	4	5
			Definitely Disagree	Somewhat Disagree	Neither Agree nor Disagree	Somewhat Agree	Definitely Agree
