# Supplementary material for: Psychometric evaluation of the Comprehensive Autistic Trait Inventory in autistic and non-autistic adults
Source: Autism. 2025 Jul 16;29(12):2955–74. doi: 10.1177/13623613251347740 (PMC12618716; doi:10.1177/13623613251347740)
Supplement: sj-zip-1-aut-10.1177_13623613251347740 – Supplemental material for Psychometric evaluation of the Comprehensive Autistic Trait Inventory in autistic and non-autistic adults [file sj-zip-1-aut-10.1177_13623613251347740.zip › CATI version changes.docx]

**CATI version changes**

**Please refer to** [**www.cati-autism.com**](http://www.cati-autism.com) **for the most current information about the CATI.**

**CATI 1.1**

Version described in *Psychometric evaluation of the Comprehensive Autistic Trait Inventory in autistic and non-autistic adults (English et al., 2025).*

Minor modifications to several sensory items and subscale names following input from an autistic advisory group

- Q4: I am **over-sensitive** to bright lighting 🡪 I am **very** sensitive to bright lighting
- Q18: I am **over-sensitive** to touch 🡪 I am **very** sensitive to touch
- Q24: I am **over-sensitive** to particular tastes (e.g., salty, sour, spicy, or sweet) 🡪 I am **very** sensitive to particular tastes (e.g., salty, sour, spicy, or sweet)
- Q36: I react **poorly** to unexpected loud noises 🡪 I react **strongly** to unexpected loud noises
- Subscale: Cognitive **Rigidity (RIG)** 🡪 Cognitive **Flexibility (FLX)**
- Subscale: **Repetitive** Behaviours **(REP)** 🡪 **Self-Regulatory** Behaviours **(REG)**

**CATI 1.0**

Original version as described in *The Comprehensive Autistic Trait Inventory (CATI): Development and validation of a new measure of autistic traits in the general population (English et al., 2021).*
